# A neural probe for concurrent real-time measurement of multiple neurochemicals with electrophysiology in multiple brain regions in vivo

**DOI:** 10.1073/pnas.2219231120

**Published:** 2023-07-03

**Authors:** Uikyu Chae, Jiwan Woo, Yakdol Cho, Jeong-Kyu Han, Soo Hyun Yang, Esther Yang, Hyogeun Shin, Hyun Kim, Hyun-Yong Yu, C. Justin Lee, Il-Joo Cho

**Affiliations:** ^a^Department of Biomedical Sciences, College of Medicine, Korea University, Seoul 02841, Republic of Korea; ^b^Research Animal Resource Center, Korea Institute of Science and Technology, Seoul 02792, Republic of Korea; ^c^Brain Science Institute, Korea Institute of Science and Technology, Seoul 02792, Republic of Korea; ^d^Department of Anatomy, College of Medicine, Korea University, Seoul 02841, Republic of Korea; ^e^School of Electrical Engineering, Korea University, Seoul 02841, Republic of Korea; ^f^Center for Cognition and Sociality, Institute for Basic Science, Daejeon 34126, Republic of Korea

**Keywords:** neural probe, biosensor, neurotransmitter, MEMS, neural circuit

## Abstract

Real-time monitoring of various neurochemicals with high spatial resolution in multiple brain regions in vivo can elucidate neural circuits related to various brain diseases. However, previous systems for monitoring neurochemicals have limitations in observing multiple neurochemicals without crosstalk in real time, and these methods cannot record electrical activity, which is essential for investigating neural circuits. Here, we present a real-time bimodal (RTBM) neural probe that uses monolithically integrated biosensors and multiple shanks to study the connectivity of neural circuits by measuring multiple neurochemicals and electrical neural activity in real time. Using the RTBM probe, we demonstrate concurrent measurements of four neurochemicals—glucose, lactate, choline, and glutamate without cross-talking each other—and electrical activity in real time in vivo. Additionally, we show the functional connectivity between the medial prefrontal cortex and mediodorsal thalamus through the simultaneous measurement of chemical and electrical signals. We expect that our device will contribute to not only elucidating the role of neurochemicals in neural circuits related to brain functions but also developing drugs for various brain diseases related to neurochemicals.

## Significance

Real-time monitoring of multiple neurochemicals has been challenging due to the crosstalk between sensors. The proposed system enables multiple measurements through the monolithically integrated microfluidic chip on a neural probe. Also, the proposed real-time bimodal neural probe allows the concurrent observation of electrophysiological signals and different neurochemical concentrations from multiple brain regions of a live animal in real time. In this manuscript, we successfully measured bimodal neural activities in vivoin the medial prefrontal cortex and the medial dorsal thalamus of an intact mouse brain. We expect that the system will contribute to not only investigating the role of neurochemicals in neural circuits related to brain function but also developing the drug for a variety of neurochemical-related brain disorders.

Monitoring various neurotransmitters in the brain can provide pivotal insights into normal brain function as well as the mechanism of various brain diseases and assist in the development of therapeutic solutions (1, 2). For example, dyskinesia in Parkinson's disease is known to be associated with various neurotransmitters, including serotonin, GABA, and glutamate (3–5). Additionally, schizophrenia is related to abnormal neurotransmission in various neural circuits, including the mesocortical, mesolimbic, and prefrontal-hippocampal pathways (6, 7). Importantly, neurochemical concentrations must be measured with high temporal resolution so that we can monitor the concentration changes induced by chemical and stimulations in real time (8). Many previous studies have measured the concentration of neurochemicals in functional neural circuits following various types of stimulation, such as chemical and optical stimulations (9, 10).

Other approaches to measuring neurochemicals have included the use of sampling devices that collect extracellular fluid (ECF) from the brain [e.g., microdialysis probe (11, 12) and push–pull cannula (13, 14)] and precise external analyzers, such as mass spectrometry (15). However, these methods showed poor spatiotemporal resolution due to the relatively large diameter of the cannula and the sampling time of several minutes necessary to collect the required sample volume for analyses (16). Recently, several devices have achieved high spatial resolution for targeting small brain regions through the microfabrication process (17). In addition, temporal resolution has been improved by analyzing extracted ECF with a separated enzymatic assay module (18, 19) or customized analytic equipment (20, 21). However, due to the large size and complicated structure of these systems, it is challenging to expand these devices to an array structure, which is essential for simultaneously observing neurochemicals in multiple regions. Moreover, to study functional neural circuits related to neurochemicals, we need to monitor neurochemicals in multiple brain regions at high spatial resolution in tandem with electrophysiology. Recently, our group reported a bimodal neural probe that can measure electrical signals while extracting ECF through push–pull perfusion (22). This system suggested the possibility of simultaneously observing electrical and chemical signals with high spatial resolution. However, it has a poor temporal resolution due to the required time of 20 min for ECF collection.

In addition, genetically fluorescence-encoded neurochemical biosensors offer an attractive, allowing for selective observation of genetically expressing neurons with excellent spatiotemporal resolution (23–26). However, these biosensors have limitations on the measurement of neurochemical concentration and the simultaneous detection of various neurochemicals due to the limited availability of light wavelengths.

As an alternative to large, complicated systems, miniaturized probes combined with enzyme-based biosensors allow neurochemicals and electrophysiological signals to be observed simultaneously by integrating a sensing electrode and a recording electrode on the tip of a probe (27). However, these systems are significantly limited in their ability to allow the observation of multiple neurochemicals with high spatial resolution due to crosstalk with neighboring biosensors attributable to the by-products generated by enzymatic reactions (28, 29). Additionally, it is difficult to integrate large sensors, which help to increase sensitivity, in these systems due to the limited size of the probe shank.

**Fig. 1.**
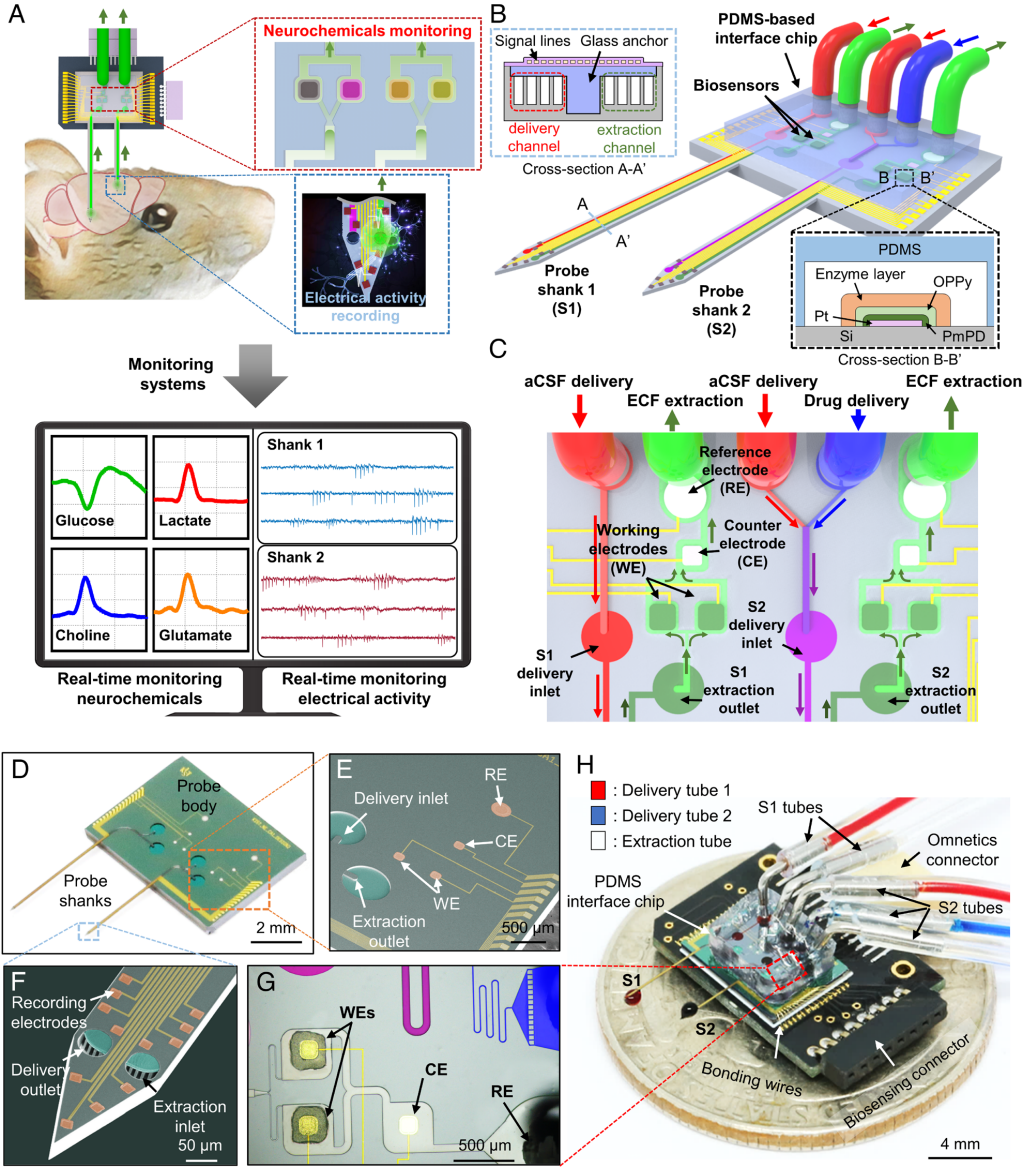
Operation principles, design, and fabrication of the multishank RTBM MEMS neural probe. (*A*) Schematic illustration of the setup for simultaneously monitoring multiple neurochemicals in real time while recording electrical neural activity using the RTBM neural probe with biosensors integrated on the probe body. The extracted ECF flows to the biosensor to enable real-time monitoring neurochemicals concentration in real time. (*B*) Schematic diagrams of the RTBM neural probe system which consists of four biosensors coated with enzyme layers, two shanks, a PDMS-based interface chip with fluid paths, and tubes for applying external pressure. The biosensor electrodes are coated with interference-blocking layers and an enzyme for selective reaction with neurochemicals and placed within a microfluidic chamber. (*C*) Schematic diagram of the probe body with three-electrode biosensors, aCSF delivery, drug delivery, and ECF extraction for real-time monitoring neurochemicals. (*D*) Optical image of the fabricated multishank RTBM MEMS neural probe. (*E*) SEM image of the biosensors and the channel inlet and outlet configured in the body of the multishank RTBM MEMS neural probe. (*F*) SEM image of a shank tip with a recording electrode array, extraction inlet, and delivery outlet. (*G*) Optical image of the biosensor array of the packaged multishank RTBM neural probe body. (*H*) Optical image of the packaged multishank RTBM MEMS neural probe.

Here, to solve the above problems, we present a real-time bimodal (RTBM) microelectromechanical system (MEMS) neural probe with monolithically integrated biosensors for simultaneously monitoring various neurochemicals and electrical neural activity in multiple brain regions in vivo. This proposed RTBM neural probe enables these multiple functions by 1) monolithically integrating enzyme-based biosensors on the probe body to simultaneously measure various neurochemicals, 2) incorporating a multishank structure that allows measurement of bimodal neural activity in multiple brain regions involved in functional connections of neural circuits to be monitored, 3) integrating microfluidic channels that extract ECF and deliver buffer solution or drugs in real time, 4) including a polydimethylsiloxane (PDMS)-based microfluidic chip that delivers extracted ECF to dedicated biosensors for each neurochemical through microfluidic channels without any crosstalk in real time and mixes multiple drugs for chemical stimulation, and 5) arranging recording electrodes near the sampling site of the probe to record electrical activity that is highly colocalized with neurochemicals (Fig. 1). In the proposed structure, we can easily increase the number of biosensors and change the enzyme species according to the target neurochemicals. Moreover, the microfluidic chip is miniaturized, and the delay in the neurochemical measurement is minimized. Consequently, our RTBM probe offers pivotal functions for investigating neural circuits by precisely measuring neurochemicals along with electrophysiological signals in real time in vivo. To demonstrate the capabilities of our RTBM probe, we show that we can simultaneously measure the concentration of four neurochemicals (namely glutamate, choline, glucose, and lactate) and electrical activity in real time in the hippocampus of a mouse. Furthermore, we successfully investigated neural circuits using two-shank RTBM neural probes by simultaneously monitoring the concentration of glutamate and glucose in real time with electrical signals in both the medial prefrontal cortex (mPFC) and mediodorsal thalamus (MD) regions, which are known to be associated with various brain diseases, such as schizophrenia.

## Results

### Operation Principles and Design of the RTBM Neural Probe.

The RTBM MEMS neural probe extracts ECF, including various neurochemicals, through a probe that is implanted into the target region of the brain and simultaneously measures the electrical activity of neurons via recording electrodes that are integrated on the probe (Fig. 1*A*). Then, the extracted ECF flows to biosensors that are integrated on the body of the probe through a PDMS-based microfluidic chip, allowing the concentration of various neurochemicals to be measured in real time. The chip has fluidic interfaces for applying external positive and negative pressure on the microfluidic channel, which is required for the push–pull operation. The proposed probe is composed of two shanks, and the lengths of each shank are designed according to the target regions to be investigated in the neural circuit (*SI Appendix*, Fig. S1). To investigate the neural circuit between the mPFC and the MD, the length of the left shank was designed as 5.5 mm (for the mPFC), and the length of the right shank was designed as 4.2 mm (for the MD). Each shank includes integrated microfluidic channels for extracting ECF and delivering drugs or artificial cerebrospinal fluid (aCSF). During the extraction process, aCSF solution is delivered to prevent the depletion of ECF in the brain. Additionally, we can deliver drugs to the target brain region to modulate neural activity.

In the proposed structure, we integrated four biosensors, which enabled the measurement of two different neurochemicals in each brain region (Fig. 1*B*). For electrochemical sensing of neurochemicals, we employed a three-electrode system and integrated an enzyme-based biosensor array with four working electrodes to monitor of redox reactions, two counter electrodes to monitor providing a current path along with working electrode, and two reference electrodes to maintain a constant potential on the body of the probe. The PDMS-based microfluidic chip includes microfluidic channels that deliver both fluids to the probe and extracted ECF to the biosensor array. The enzyme-coated working electrodes were located inside individual chambers, and the chamber was located in parallel to prevent crosstalk among the sensors. Furthermore, the length of the fluidic paths from the probe to the sensors was designed to shorten the delay between the extraction and measurements (Fig. 1*C*). In addition, we incorporated channel resistance patterns into each chamber to allow the extracted ECF to flow uniformly into each biosensor chamber in parallel (*SI Appendix*, Fig. S2). The reference electrodes were coated with silver/silver chloride (Ag/AgCl) ink and located near the extraction ports, and the counter electrodes were located between the working electrode and the reference electrode. The size of the probe shank was minimized by completely embedding the microfluidic channels within the shank, as previously reported (22).

The proposed RTBM MEMS neural probe is designed with a small form factor for experiments with mice by monolithically integrating biosensors, which enable multiple neurochemicals to be monitored in real time without crosstalk while simultaneously recording electrical activities. In addition, the two-shank RTBM neural probe, which was designed for investigating neural circuits, allows real-time observation of concurrent bimodal neural activity in multiple brain regions. Additionally, the design of the probe can easily be reconfigured according to the target brain region and neurochemicals. For our in-depth study on the neural activities related to various neurochemicals in specific brain regions, we integrated four biosensors into the body of a probe with a single shank (*SI Appendix*, Fig. S3).

### Fabrication and Packaging of the RTBM MEMS Neural Probe.

We fabricated the RTBM MEMS neural probe with a microfabrication process that enables not only miniaturized shanks that minimize tissue damage but also a small form factor that allows the system to be applied to mice and bimodal neural signals to be recorded in real time The size of the working electrode, which determines the sensitivity of the biosensor, was maximized as 150 μm × 150 μm, with consideration of a sufficient margin for the packaging, and the counter and reference electrodes had 400 μm diameters (Fig. 1*E*). Twelve recording electrodes (19 μm × 19 μm), the inlet of the extraction channel, and the outlet of the delivery channel (diameter of 52 μm) were located within 200 μm of the probe tips (Fig. 1*F*). In addition, the probe shank with the delivery and extraction channels included five individual 15 μm (height) × 7 μm (width) microfluidic channels on each side, with an anchor placed at the center. The width and height of the probe were 144 μm and 40 μm, respectively.

On the body of the fabricated RTBM neural probe, we integrated a multiple enzyme-based biosensor. First, the fabricated RTBM neural probe was attached to a customized printed circuit board (PCB) to facilitate handling of the device, and the pads of the probe were electrically connected to the pads of the PCB through wire bonding. Then, we coated Ag/AgCl ink on the previously patterned Pt electrode, as the reference electrode (RE). Then, the working electrodes were coated with interference-blocking layers of poly(meta-phenylenediamine) (PmPD) and overoxidized polypyrrole (OPPy) by electropolymerization. The PmPD layer is an excellent interference-blocking layer for blocking electroactive interferents, such as ascorbic acid (AA) and dopamine (DA), which interfere with the measurement of target neurochemicals and selectively react with enzymes (30). However, since the PmPD layer is prone to damage during the subsequent packaging procedure, which includes O_2_ plasma treatment (31), we deposited an OPPy layer as a protective layer that can be deposited with a thickness of several micrometers (30).

In the packaging process, the most challenging procedure is the selective enzyme coating process on each working electrode. Due to the small distance between the working electrodes, if the enzyme solution, composed of the enzyme, bovine serum albumin (BSA), glutaraldehyde (GA), and glycerol, is added dropped, the solution might overflow to a neighboring working electrode and degrade the selectivity of the biosensor. Thus, we fabricated a customized PDMS-based well array, which was temporarily attached to the working electrode array to confine the enzyme solution. The well size was designed to be 250 μm × 250 μm, which is slightly larger than the size of the working electrode (150 μm × 150 μm). Then, the enzyme solution was added dropped on each well, and we waited 1 h at room temperature for the enzyme layer to be sufficiently cross-linked. We coated different enzyme layers on each electrode selectively to monitor various neurochemicals, after which the well array was removed. The proposed enzyme coating process allowed the integration of four enzyme-based sensors in a small probe body. Next, we permanently bonded a PDMS-based interface chip engraved with microchannels through the SU-8 master mould (32) on the probe body through an O_2_ plasma treatment (Fig. 1*G* and *SI Appendix*, Fig. S4). For the interface with measurement equipment, we attached an Omnetics connector for electrical connections and connected plastic tubes for fluidic connections (Fig. 1*H*). Finally, the recording electrode array and biosensors were connected to external amplifiers, and positive or negative pressure was applied to each fluidic tube to facilitate push–pull operations (*SI Appendix*, Fig. S5).

### Characterization of Biosensors Integrated on the RTBM MEMS Neural Probe.

To measure various neurochemicals simultaneously, we need to not only remove any crosstalk but also characterize the sensitivities of the sensors. Additionally, the flow rate should be optimized to reduce the time delay between the electrical and chemical signals while ensuring stable push–pull perfusion. Therefore, before we applied the RTBM MEMS neural probe to in vivo experiments, we characterized the crucial functionalities of our RTBM neural probe.

To measure the neurochemical concentrations in real time, we needed to generate continuous brain fluid flows through push–pull operations. Thus, we applied negative pressure on the extraction channel of the interface chip to generate a continuous flow of extracted brain fluid and positive pressure on the delivery channel to deliver aCSF to the brain. The sensitivity of the biosensor was inversely proportional to the flow rate (33) (*SI Appendix*, Fig. S6). Therefore, it was confirmed that the flow rate should be constantly maintained to ensure accurate measurements. By controlling the applied pressures, we optimized the flow rate to 100 nL min^−1^ which was verified in previous work (22) by applying a positive pressure of 18.5 kPa and a negative pressure of −16.5 kPa.

**Fig. 2.**
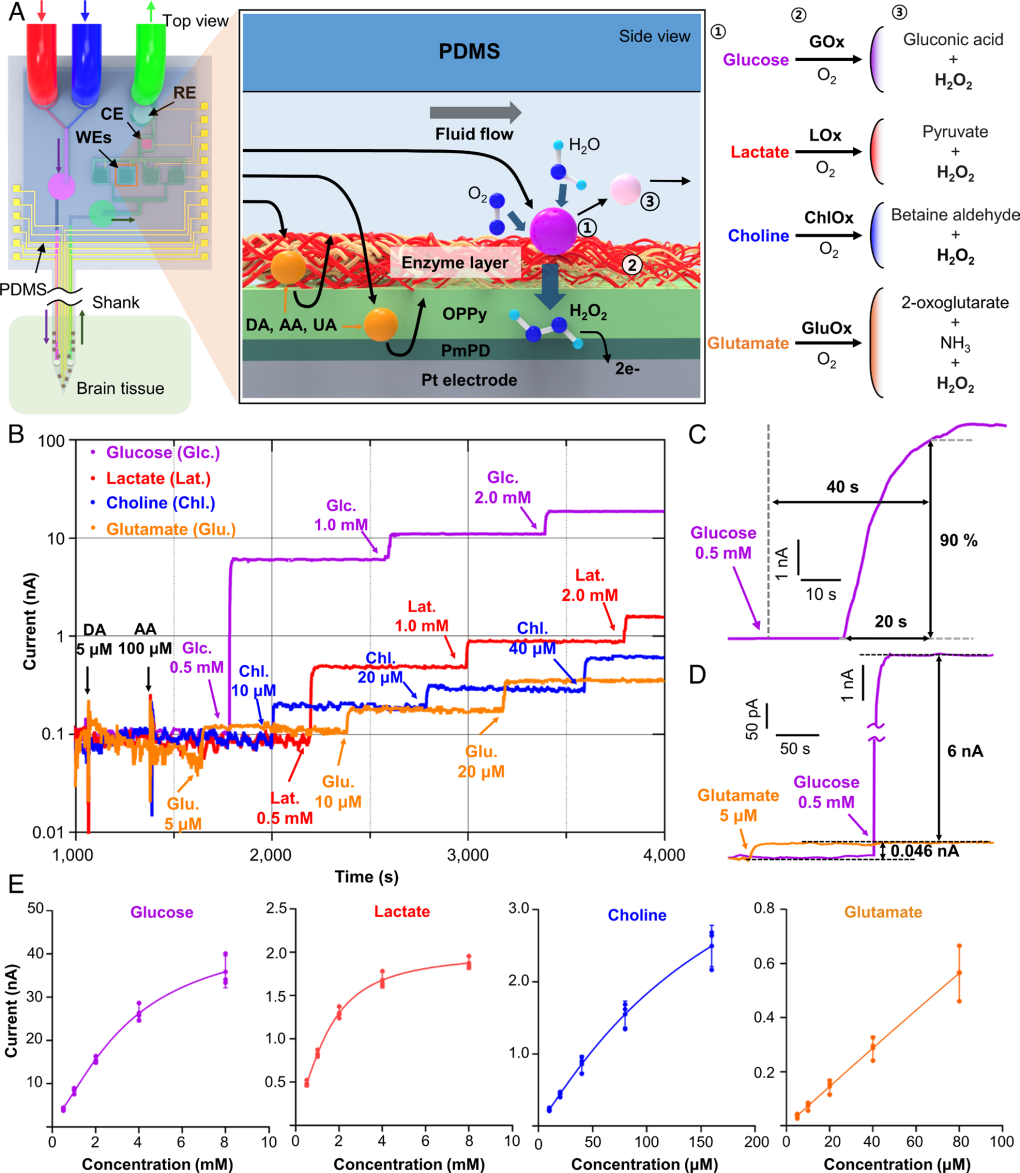
Characterization of biosensors integrated on the RTBM MEMS neural probe. (*A*) Schematic diagram illustrating the sensing mechanism of neurochemicals using enzyme-based biosensors that are monolithically integrated on the probe body. Hydrogen peroxide generated by the reaction of oxidase and neurochemicals on the biosensor electrode is oxidized by the potential of the Pt electrode to generate an electric current. By selecting the type of oxidase according to neurochemicals, it is possible to selectively observe the concentration of neurochemicals. (*B*) Oxidation current responses of the corresponding biosensors for glucose (purple), lactate (red), choline (blue), and glutamate (orange) as a function of the concentrations after injecting interferents. (*C*) Response time of the glucose biosensor after injection of 0.5 mM glucose and saturation time when 90% of the oxidation current was compared to the saturated current. (*D*) Evaluation of crosstalk between the glucose and glutamate biosensors via observation of oxidation currents. (*E*) Calibration curves showing the sensitivities of glucose, lactate, choline, and glutamate.

We next investigated the ability of the biosensors to measure the concentration of multiple neurochemicals in real time under this precisely controlled fluid flow. Fig. 2*A* shows the sensing mechanism of multiple neurochemicals in real time with enzyme-based biosensors integrated on the probe body. In this experiment, we selected four target neurochemicals that could represent changes in the neural activities of target brain regions: glucose, a major energy source for cells (34); lactate, an important metabolite in the nervous system (35); glutamate, a representative excitatory neurotransmitter (36); and choline, another excitatory neurotransmitter that is directly related to acetylcholine (37).

To measure the sensitivity of the biosensors in an environment mimicking an in vivo experiment, we immersed the probe tip in a beaker filled with aCSF and added the target neurochemicals at various concentrations and the electroactive interferents DA and AA and extracted the aCSF. Additionally, during the experiment, we applied a working potential of 0.6 V vs. an Ag/AgCl reference electrode to four working electrodes and maintained the flow rate at 100 nL min^−1^, which enabled continuous flow of the extracted aCSF, and we measured the current from four biosensors while adding various concentrations of glutamate, glucose, choline, and lactate to the aCSF-filled beaker. The current began to change only 20 s after the target neurochemicals were added to the beaker due to the small volume of the microfluidic channel (Fig. 2*B*), which implies that there is a timing difference between electrical neural signals and neurochemical concentrations. Additionally, the biosensor required 20 s to reach 90% of the final current value, which indicates that the temporal resolution of the sensor was 20 s (Fig. 2*C*).

Next, we determined whether there was any crosstalk between the biosensors by the H_2_O_2_ generated by the enzyme reaction. We added 0.5 mM glucose and measured the current of the other biosensors; no current change was measured, whereas a current change of 17.6% was measured with the serially arranged sensors (*SI Appendix*, Fig. S7), confirming that the parallel arrangement of the biosensors is effective for preventing crosstalk (Fig. 2*D*).

Finally, to show the reliability of the fabrication process, we measured the sensitivity of four different biosensors from three different neural probes (Fig. 2*E*). For the glucose sensors, the sensitivity was measured to be 6.18 ± 0.71 nA mM^−1^ below 4 mM (R^2^ = 0.9679), while the lactate sensors showed a sensitivity of 0.62 ± 0.07 nA mM^−1^ below 2 mM (R^2^ = 0.9767). The normal glucose and lactate concentrations in the rodent brain are known to be within the ~2.5 mM range (38, 39). Thus, our biosensors could sufficiently measure the concentrations of lactate and glucose in the linear range of up to 2 mM, and concentrations above this range can also be estimated. For the glutamate and choline sensors, the sensitivities were measured to be 7.03 ± 1.26 pA μM^−1^ (glutamate) and 19.82 ± 1.09 pA μM^−1^ (choline), and the sensitivity was linear up to 80 μM, with R^2^ = 0.9642 (glutamate) and 0.9779 (choline). The concentrations of glutamate and choline in the rodent brain are within the μM range; thus, our biosensors can measure these neurotransmitters sufficiently within a linear range (9, 40). In addition, we calculated the detection limit of the glutamate and choline sensors to be 0.29 μM and 0.14 μM, respectively.

Finally, we conducted experiments to examine the potential cross-talk of the fabricated sensors with neurochemicals that have similar structures to their target molecules. Specifically, we tested the glucose sensor with fructose and galactose; the lactate sensor with pyruvate and malate; the choline sensor with acetylcholine and ethanolamine; and the glutamine sensor with glutamine and aspartate. We found that the glucose sensor generated a negligible 1.6% of current to galactose compared to glucose (41), while the other sensors did not show any cross-talk to neurochemicals with similar structures (*SI Appendix*, Fig. S8).

The sensitivity, linearity, and detection limit results affirm that the performance of the biosensors was sufficient for simultaneously measuring concentration changes for multiple neurochemicals in various brain regions in real time.

### Sensitivity of Biosensors in Response to Electrical Stimuli Ex Vivo.

Before conducting in vivo experiments, we characterized the sensitivity of the sensors ex vivo in acute slices where we could well control physiological parameters such as pH and temperature. The fluidic channels integrated in the system were filled with aCSF to ensure that no air bubbles were present and to stabilize the biosensor before the experiment. The neural probe was promptly inserted into the hippocampal CA3 region of the brain slice by using the recording electrodes on the probe tip as anode and cathode electrodes for electrical stimulation (*SI Appendix*, Fig. S9*A*). Initially, we measured the baseline concentrations of three neurochemicals (lactate = 130.9 μM; choline = 1.7 μM; and glutamate = 2.4 μM) excluding the glucose sensor due to the high concentration of glucose (11 mM) in the aCSF. Then, we measured the change in neurochemical concentrations induced by electrical stimulation. During the experiment, we monitored the concentration changes of neurochemicals triggered by electrical stimulation in the CA3 region (*SI Appendix*, Fig. S9*B*). We applied three stimuli with 5-min intervals (*SI Appendix*, Fig. S9*C*) and statistically analyzed the measured values (*SI Appendix*, Fig. S9 *D*–*F*). As a result, the statistical analysis confirmed that electrical stimuli significantly increased the concentrations of all three neurochemicals. The maximum concentration of lactate concentration increased by 16.6%, choline by 92.9%, and glutamate by 128.3%, respectively. Therefore, we confirmed that the fabricated RTBM neural probe has enough sensitivity to detect the concentration change of neurochemicals induced by changes in neural activity.

### Real-Time Monitoring of Bimodal Neural Activity with Neuromodulation In Vivo.

To show the functionality of the probe, we measured the concentration of multiple neurochemicals (glutamate, choline, glucose, and lactate) and electrical signals in the hippocampal region of a live mouse stimulated with a high concentration of KCl. Previous studies have reported that changes in neural activity induce concentration changes in lactate and glucose (41), which would enable monitoring of neural activity. By comparing electrical signals with concentration changes in glucose and lactate, we can confirm the functionality of monitoring glucose and lactate concentrations with our proposed system.

Furthermore, cholinergic neurons and glutamatergic neurons are known to be abundant in the hippocampal region (42), and changes in the concentration of choline and glutamate allow us to monitor changes in the activity of cholinergic and glutamatergic neurons, which are difficult to obtain from electrical signals. Therefore, monitoring bimodal neural activities in vivo is a stepping stone for precisely understanding the nervous system and studying neural circuits in-depth.

**Fig. 3.**
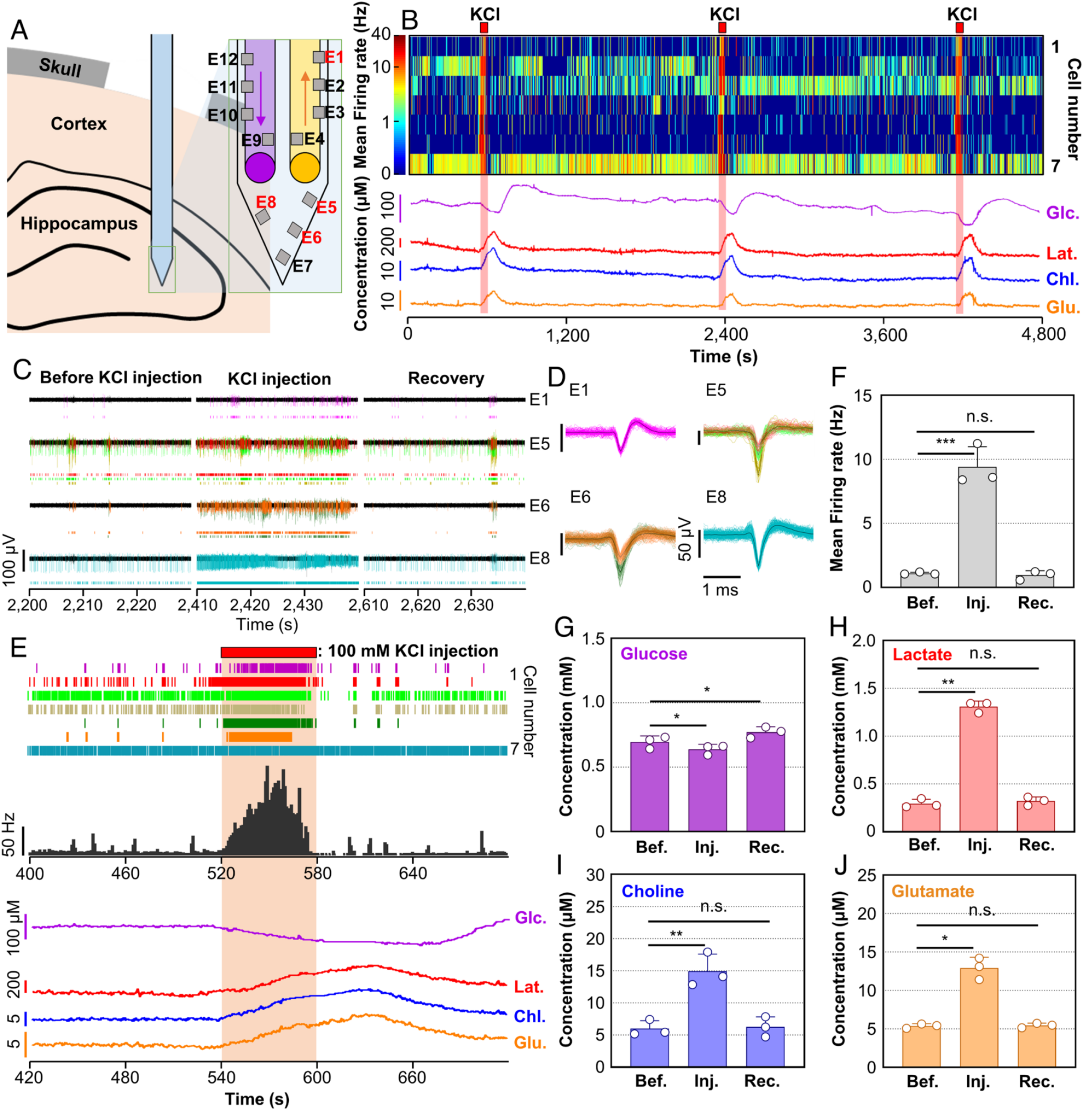
In vivo experimental results for real-time monitoring of four neurochemicals and electrical activity with the one-shank RTBM MEMS neural probe. (*A*) Schematic illustration of the one-shank RTBM neural probe inserted into the CA3 of the mouse hippocampus, showing a schematic of the electrode numbers on the probe tip. (*B*) Heatmap showing changes in the firing rates of neural signals recorded on representative electrodes E1, E5, E6, and E8 after KCl injection. Response curves showing the concentrations of four neurochemicals (glucose, lactate, choline, and glutamate) upon KCl injection. (*C*) Transient plots of electrical signals recorded before, during, and after (recovery) KCl injection. (*D*) Waveforms of the sorted spikes from the transient plots. (*E*) Raster plots and firing rates of the recorded electrical signals and variation in the neurochemicals measured before, during, and after KCl injection. (*F*–*J*) Changes in firing rates and concentrations of the four neurochemicals measured before, during, and after KCl injection: (*F*) mean firing rate, (*G*) glucose, (*H*) lactate, (*I*) choline, and (*J*) glutamate. In *F*–*J*, data are presented as the mean ± SD (n = 3 for all, two-tailed *t* test). In *F*, ***P = 0.0008, *t* = 9.111 [Before (Bef.) – Injection (Inj.)]; *n.s.* = 0.5286, *t* = 0.6893 [Bef. – Recovery (rec.)]. In *G*, *P = 0.0192, *t* = 7.107 (Bef. – Inj.); *P = 0.0421, *t* = 4.718 (Bef. – Rec.). In *H*, **P = 0.0011, *t* = 29.69 (Bef. – Inj.); *n.s.* = 0.1674, *t* = 2.126 (Bef. – Rec.). In *I*, **P = 0.0084, *t* =10.84 (Bef. – Inj.); *n.s.* = 0.5627, *t* = 0.6877 (Bef. – Rec.). In *J*, *P = 0.0143, *t* = 8.287 (Bef. – Inj.); *n.s.* = 0.6507, *t* = 0.5271 (Bef. – Rec.).

First, we moved the fluid continuously at a flow rate of 100 nL min^−1^ to remove all the bubbles in the extraction channel of the PDMS interface chip and probe. Bubbles must be removed from the microfluidic channels to continuously monitor the neurochemicals. Then, the biosensors were stabilized in the fluid flow by applying a potential of 0.6 V vs. an Ag/AgCl reference electrode on each working electrode. The RTBM neural probe was implanted in the hippocampal region (coordinates: AP −1.70, ML −1.70, DV −1.80; millimeter, relative to bregma) of a mouse. and we matched a constant sampling flow rate of 100 nL min^−1^ for 30 min. Then, we simultaneously recorded the electrical activity and measured the neurochemical concentrations while modulating neural activity by injecting the KCl solution (i.e., 100 mM KCl in aCSF) at 30-min intervals (Fig. 3*B*). We confirmed that the electrical signals recorded by four representative electrodes showed marked increases in electrical activity during KCl injections and then recovered to the previous state after the injected solution was switched to aCSF (Fig. 3 *C* and *D*). Additionally, When the KCl solution was injected, the electrical signals changed, followed by changes in the neurochemicals after a short delay of 20 s. The glucose concentration temporarily decreased, whereas the lactate, choline, and glutamate concentrations increased, as expected based on previous works (9, 43) (Fig. 3*E* and *SI Appendix*, Fig. S10).

We also compared the changes in the bimodal neural signals before and during injection of the KCl solution and recovery through three repetitions of chemical stimulation to evaluate the reliability of our RTBM neural probe. KCl injection induced a dramatic increase in the firing rates of neurons, with an average increase of 548.6%, and after switching to aCSF, the firing rates returned to prestimulation levels (Fig. 3*F*). When we compared the change in firing rate with KCl injection for each cell, we confirmed that all cells showed an increase in firing rate with KCl injection (*SI Appendix*, Fig. S11). Furthermore, a comparison of the neurochemical concentrations before, during, and after KCl injection showed significant changes that were similar to changes in the electrical activity. In particular, the glucose concentration decreased by an average of 8.2% during KCl injection; however, the glucose concentration returned to levels that were an average of 11.0% higher than the levels before KCl injection (Fig. 3*G*). It is known that the decrease in glucose concentration caused by this stimulation is due to the maintenance of homeostasis by increasing the activity of cells (44). In addition, during KCl injection, the concentrations of glutamate, choline, and lactate increased by 140.0%, 148.8%, and 236.3%, respectively, and after switching to aCSF, the concentrations returned to the initial levels before KCl injection (Fig. 3 *H*–*J*). Therefore, we successfully confirmed the functionalities of our RTBM neural probe as a tool for investigating brain functions by showing that our probe could measure the concentrations of four neurochemicals in conjunction with electrical neural signals in target brain regions in real time.

Additionally, we conducted an experiment to demonstrate the selectivity of our system in measuring neurochemicals, as suggested by the reviewer. For this purpose, we utilized DL-three-β-benzyloxy aspartate (DL-TBOA), a reuptake inhibitor that specifically modulates glutamate levels. Following the same experimental protocol and conditions as before, we implanted the probe into the hippocampus and measured the neurochemical concentrations. The results show that DL-TBOA injection (1 mM, 1 min, 100 nL) selectively increased the concentration of glutamate from 4.4 μM to 10.5 μM, a 138% increase. On the other hand, the concentrations of choline, glucose, and lactate did not exhibit a significant difference following DL-TBOA injection (*SI Appendix*, Fig. S12). This experiment confirms the selectivity of our biosensor system and its capability for the simultaneous monitoring of various neurochemicals.

### Investigation of Neural Circuits through Real-Time Monitoring of Bimodal Activities in the mPFC and MD Regions.

Given the importance of examining the role of specific neurons in neural circuits, we aimed to measure the activity of specific neurons by measuring the concentrations of neurotransmitters related to that cell type. Specifically, we aimed to evaluate the glutamatergic neurons in the neural circuit between the mPFC and MD regions, which is known to be associated with schizophrenia (45), by measuring the glutamate concentration and electrical activity in both regions.

**Fig. 4.**
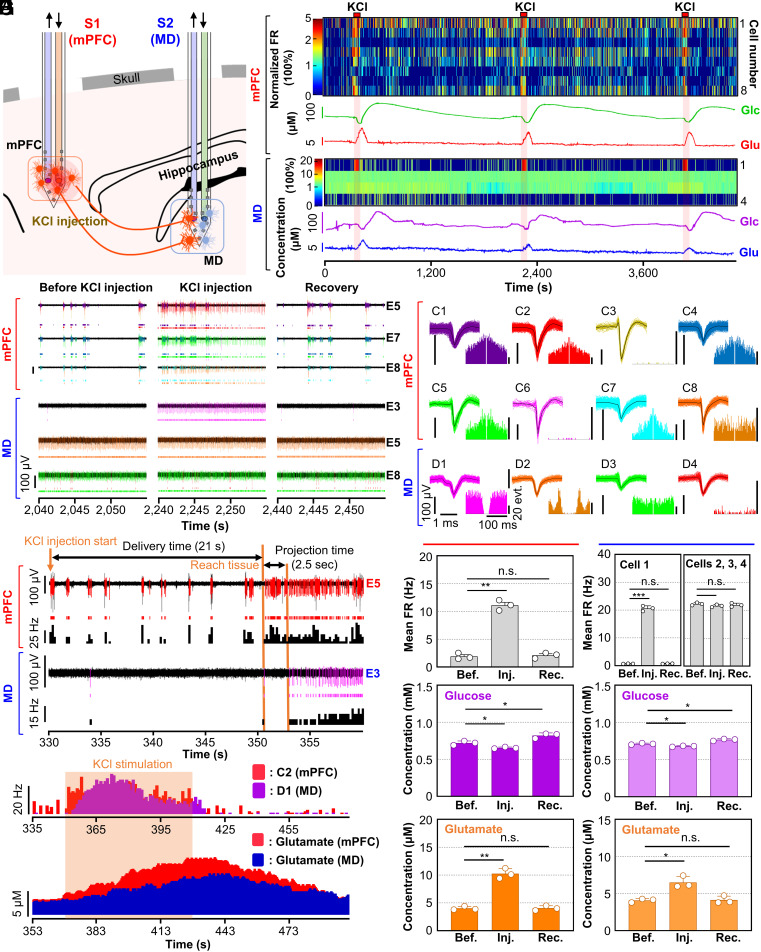
Investigation of functional connectivity through monitoring of neurochemicals and recording electrical signals. (*A*) Schematic of the RTBM MEMS neural probe inserted in the mPFC and MD in the intact brain of a mouse. KCl injection into the mPFC-induced neuromodulation for the investigation of functional connectivity. (*B*) Heatmaps showing changes in the firing rates of neural signals recorded by electrodes in shank 1, which was inserted in the 3mPFC, and shank 2, which was inserted in the MD, after KCl injection (the mean firing rates calculated before KCl injection were normalized to 100%). Response curves showing the concentrations of glucose and glutamate in the mPFC and MD after KCl injection. (*C*) Transient plots of electrical signals recorded in the mPFC and MD before, during, and after (recovery) KCl injection. (*D*) Representative transient plots with raster plots of sorted electrical spike signals and firing rates of electrical signals from representative transient plots in the mPFC and MD after KCl injection. (*E*) Firing rates of representative electrical signals and variations in the measured glutamate concentration in the mPFC (red) and MD (blue and purple) during and after KCl stimulation in the mPFC. (*F*) Waveforms of the sorted spike signals with autocorrelation histograms from transient plots. (*G*–*I*) Changes in the firing rates and concentrations of glucose and glutamate measured in the mPFC (*Left*) and MD (*Right*) before, during, and after KCl injection in the mPFC: (*G*) firing rates, (*H*) glucose, (*I*) glutamate. In *G*–*I*, data are presented as the mean ± SD (n = 3 for all, two-tailed *t* test); In *G*, *Left*, **P = 0.0053, *t* = 13.76 (Bef. – Inj.); *n.s.* = 0.3568, *t* = 1.1188 (Bef. – Rec.); *Right*, ***P = 0.0008, *t* = 35.08 (Bef. – Inj. in cell 1); *n.s.* = 0.5892, *t* = 0.6372 (Bef. – Rec. in cell 1); *n.s.* = 0.2814, *t* = 1.461 (Bef. – Inj. in cells 2, 3, and 4); *n.s.* = 0.5903, *t* = 0.6352 (Bef. – Rec. in cells 2, 3, and 4). In *H*, *Left*, *P = 0.0230, *t* = 6.476 (Bef. – Inj.); *P = 0.0112, *t* = 9.351 (Bef. – Rec.); *Right*, *P = 0.0165, *t* = 7.686 (Bef. – Inj.); *P = 0.0490, *t* = 4.350 (Bef. – Rec.). In *I*, *Left*, *P = 0.0029, *t* = 18.41 (Bef. – Inj.); *n.s.* = 0.7462, *t* = 0.3712 (Bef. – Rec.); *Right*, *P = 0.0225, *t* = 6.552 (Bef. – Inj.); *n.s.* = 0.854, *t* = 0.2083 (Bef. – Rec.).

After air bubbles were removed from the extraction channel and the biosensors were stabilized, the probe array was inserted into the mPFC (coordinates: AP 1.70, ML −0.35, DV −1.85; millimeter, relative to bregma) and the MD (coordinates: AP −1.20, ML −0.35, DV −3.15; millimeter, relative to bregma). We then modulated neural activity in the mPFC region by injecting KCl solution for 1 min at 30-min intervals (Fig. 4*A*). We observed a change in the neural activity in the mPFC region 21 s after starting the KCl injection. Interestingly, chemical stimulation in the mPFC region not only induced activity changes in the mPFC region but also modulated neural activity in the MD region (Fig. 4 *B* and *C*). During the experiment, this time delay was expected due to the delivery of KCl through the microfluidic channel, similar to the previous experiment. After neurons in the mPFC region reacted to KCl stimulation, we observed a change in the neural activity of the MD region within 2.5 s (Fig. 4*D*). Considering the physical distance of 3.3 mm between the mPFC and the MD regions, we inferred that the instantaneous change in the MD region was induced not by the diffusion of KCl but by the chemical stimulation in the mPFC region due to the functional connectivity between the two regions.

Specifically, only one neuron out of 4 neurons recorded in the MD region showed a significantly increased firing rate after KCl stimulation, while 7 out of 8 recorded neurons in the mPFC showed an increased firing rate. We speculated that some neurons in the MD region are functionally connected to mPFC neurons through a glutamatergic pathway (Fig. 4*E*). Accordingly, the glutamate concentration in the mPFC region increased 18 s after the firing rate in the mPFC region increased due to the required time to deliver the ECF to the biosensors, as expected. Furthermore, we adjusted for the variation in extraction time by calculating the difference in fluidic channel volumes between the two shanks, which we found to be 1.3 nL. This disparity resulted in a 1 s in the observed change in the MD region compared to that in the mPFC (*SI Appendix*, Fig. S13). The glutamate concentration in the MD region also increased. However, the concentration change in the MD region was lower than that in the mPFC region because the number of activated glutamatergic neurons was smaller in the MD region than in the mPFC, as we observed from the recorded electrical signals. To analyze the recorded signals, we sorted individual spikes and calculated the autocorrelation of the sorted clusters, which confirmed that we could record signals from multiple neurons in both brain regions (Fig. 4*F*).

Additionally, we examined the cross-correlation of detected events between the MD and mPFC regions. Our investigation showed that during the three KCl injections, the average cross-correlation value between the change of MD neuron 1(D1)’s firing rate and the mPFC neurons’ firing rate was as high as 0.696, whereas the average cross-correlation values between the change of D2, D3, and D4 neurons firing rates and the change of mPFC neurons’ firing rates were very low as 0.005, 0, and 0.07, respectively. Before KCl injection, the average correlation values between MD neurons’ firing rate change and mPFC neurons’ firing rate change were all below 0.02 (D1: 0.0054, D2: 0.0017, D3: −0.0022, D4: 0.0197) (*SI Appendix*, Fig. S15). Therefore, we can conclude that the activity change of D1 neuron during KCl injection is highly correlated to the change of neural activity in mPFC neurons. Our observation suggests that only the D1 neuron among the recorded neurons was associated with mPFC activity, as not all neurons in the MD region are supposed to be connected to the mPFC.

In this experiment, we chemically stimulated neurons in the mPFC three times and measured both electrical and chemical activity changes to confirm the reliable operation of our RTBM probe. Neurons in the mPFC and MD regions were stimulated by KCl injection, and we observed significant changes in the concentrations of glutamate and glucose in both regions. Compared to spontaneous signals, the average firing rate of the stimulated neurons in the mPFC region was increased by 482.9%, which was similar to the results of our previous in vivo experiment using the one-shank RTBM neural probe. In the MD region, the firing rate of one neuron was significantly increased by 2772.6% after chemical stimulation of the mPFC region (Fig. 4*G*). However, the firing rates of other neurons did not show any significant differences. During the repetitive chemical stimulations, the glucose concentrations in the mPFC and MD regions were decreased by 9.1% and 4.5%, respectively, and then increased by 14.0% and 7.8% after KCl injection (Fig. 4*H*). Furthermore, we confirmed that during KCl injection, glutamate concentrations increased by 154.3% and 59.4% in the mPFC and MD regions, respectively, and then returned to normal glutamate concentrations after KCl injection (Fig. 4*I*). To confirm the mPFC-MD neural circuit and provide the structural connectivity between the two regions, we performed viral tracing experiments. GFP expression was then confirmed in both the AAV-injected mPFC region and the projected MD region (*SI Appendix*, Fig. S16). These results sufficiently indicate that our two-shank RTBM neural probe is a powerful tool for investigating neural circuits through the direct observation of bimodal neural activities in functional neural circuits.

## Discussion

We have described the development of a neural probe that is capable of simultaneously measuring the concentrations of multiple neurochemicals and recording electrical activity in multiple brain regions in vivo in real time. Furthermore, the proposed structure not only provides excellent expandability by increasing the number of biosensors or shanks but also permits easy reconfiguration of target neurochemicals by replacing the enzymes corresponding to the neurochemicals to be observed. When compared to a neural probe that includes biosensors on a shank, our system enables larger sensors, improving the sensitivity and eliminating crosstalk among the sensors. The proposed system also incorporates various functions, including drug delivery and electrical signal recording, in a compact shank; thus, our system can be applied to small animals, such as mice. The microfluidic chip integrated on the body allows the system to have a small form factor, which is beneficial for practical applications in experiments with small animals such as mice.

In this study, we demonstrated the capabilities of our RTBM neural probe for monitoring bimodal neural activity in real time through in vivo experiments, enabling us to correlate four neurochemical concentrations with electrical activities. Moreover, we verified the powerful potential of our RTBM neural probe by monitoring the bimodal activity in a functional neural circuit using the multishank structure. We verified that the glutamate concentration increased in both regions when the glutamatergic pathway was activated. This study is the first experiment to directly investigate the glutamatergic pathway by monitoring bimodal neural activity in real time.

Of note, it would also be possible to reveal the role of specific types of neurons in unknown neural circuits by monitoring the concentration of neurotransmitters related to these types of neurons using this neural probe. Moreover, the multidrug delivery would enable delivery of target drug and monitoring of drug efficacy through the measurement of bimodal neural activities (46).

In addition, in our system, the biosensors are monolithically integrated on the probe body, which allows various detection methods to be used to monitor neurochemicals. In addition to enzyme-based biosensors, antigen-antibody reactions (47) can be applied to our biosensors to directly identify important factors related to brain diseases. Furthermore, by integrating photodiode sensors (25) on the probe body, our system can be extended to observe a variety of neurochemicals through fluorescent enzymatic assays. Thus, we can choose any type of biosensor according to the target neurochemicals and biomarkers, maximizing the performance of our system.

During the measurement of neurotransmitters in vivo, we sometimes observed a drift in the baseline current due to equipment instability. Although the amount of drift is less than the concentration change caused by KCl injections, it needs to be suppressed because it may introduce an error in the measurement of basal concentration levels. Also, our system is specifically designed to detect neurochemicals that are released and diffused through KCl injection or electrical stimulation. Hence, it enables the measurement of concentration changes in neurotransmitters with electrical signals resulting from changes in neural activity within a specific brain region. This capability is particularly useful for experiments that necessitate direct observation of the relationship between neural activity and particular neurochemicals (48). By integrating optical stimulation capability, it would be possible to improve the temporal resolution of stimulation in smaller regions. As the response time of the sensor is influenced by the diffusion of neurochemicals, reducing the stimulated region and duration of stimulation may help to enhance the response time of the chemical sensor to the change in neurochemical concentration.

Additionally, more fundamental questions about the brain could be addressed through additional functional expansions of our system. One potential improvement would be the concurrent monitoring of bimodal signals in behaving animals. Specifically, monitoring the link between behaviors and neural activity, including the concentrations of cause substances, is of great importance in the investigation of brain diseases in disease model animals. Therefore, brain research can be radically advanced by observing the electrical and chemical neural activities of behaving animals through packaging of the proposed system in a small form factor including flexible fluidic tube. The integration of various stimulation capabilities within the small packaging would enable the investigation of neural circuit with physiologically relevant stimuli in behaving animal. Also, the system can be developed to a wireless system by integrating wireless hydrolysis micropumps and wireless recording systems.

## Materials and Methods

### Materials and Chemicals.

We used aCSF as buffer solution that contained 126 mM NaCl, 24 mM NaHCO_3_, 1 mM NaH_2_PO_4_, 2.5 mM KCl, 2.5 mM CaCl_2_, and 2 mM MgCl_2_. The pH of the buffer solution was 7.4. Monosodium glutamate, sodium lactate, choline chloride, glucose, ascorbic acid, and dopamine hydrochloride were used for the characterization of our biosensors. As enzymes, glutamate oxidase (Yamasa Corporation, Japan), lactate oxidase from Aerococcus viridans, choline oxidase from *Alcaligenes* sp., and glucose oxidase from *Aspergillus niger* were used. Chloroplatinic acid hydrate, 0.1 M HCl, and lead acetate trihydrate were used for the Pt black electroplating on the recording electrode. All reagents and materials unless otherwise noted were purchased from Merck (USA). Glutaraldehyde (50 % in water) and bovine serum albumin (BSA) were contained in the enzyme solution, and meta-phenylenediamine (mPD) and pyrrole (Py) for the interference-blocking layer were also purchased from Merck (USA). Ag/AgCl ink (Cat. No. 011464) to be used as a reference electrode was obtained from ALS Co., Ltd (Japan).

### Preparation of Biosensor Electrodes.

We prepared monolithically integrated biosensor electrodes in the probe body to observe various neurochemicals. First, we attached the fabricated neural probe on a PCB to facilitate handling of the device and provide electrical connections. Then, we coated Ag/AgCl ink on the reference electrode and cured it in an oven at 80 °C for 30 min. Afterward, we cleaned the working electrodes electrochemically by scanning a potential in the range of −0.6 to 1 V at 100 mV s^−1^ in 0.1 M phosphate-buffered saline (PBS). A PmPD layer as an interference-blocking layer was electropolymerized on working electrodes with 20 mM mPD in deionized (DI) water at 0.25 to 0.75 V for 60 cycles, a scan rate of 50 mV s^−1^. Additionally, we carried out electropolymerization of the PPy layer on the PmPD-coated electrodes with 200 mM pyrrole in 0.1 M PBS at pH 7.4 by holding the potential at 0.85 V with the reference to an Ag/AgCl for 30 s. Then, we overoxidized PPy at a constant potential at 0.85 V in 0.1 M PBS at pH 7.4 for 2 h until the steady-state current was recorded. All chemical processes for preparing the biosensor electrodes were carried out in a two-electrode system using a reference electrode coated with Ag/AgCl ink after forming the solution as a droplet on the probe body.

### Preparation of a PDMS-Based Well Array for Selectively Enzyme Layer Coating.

We fabricated a PDMS-based well array to coat the enzyme layer that selectively reacts to a target neurochemical only on the working electrode. First, the PDMS solution mixed with the base to curing agent in a ratio of 10:1 was poured into a duralumin-based master mold with a 100-μm depth cavity. The duralumin master mold filled with mixed PDMS solution was placed in an 80 °C oven for 2 h for curing the PDMS solution. After the completely cured PDMS piece was released from the master mold, Teflon tape was attached to the PDMS piece. Teflon tape prevents the shape of the PDMS from being damaged by too much heat during the laser cutting process. We patterned four holes with 250 μm × 250 μm using a laser cutter (Speedy 1000, Trotec laser, inc., Austria) on the cured PDMS piece. Laser cutting conditions were power 100, speed 3.00, PPI/Hz 3,000 Hz. Finally, we removed the Teflon tape from the PDMS-based well array and cleaned it through sonication in ethanol for 20 min.

### Enzyme Layer Patterning.

We selectively coated the enzyme layer on each working electrode using the fabricated PDMS-based well array. First, we aligned a PDMS-based well array and the biosensor array under the microscope and temporarily attached a well array on substrate. Then, we treated the PDMS-wall attached probe with O_2_ plasma treatment (100 W, 50 mTorr for 20 s, Covance-MP, Femto Science, Korea) to ensure that the enzyme solution was uniformly distributed on the working electrode. Next, we selectively added dropped 0.5 μl of enzyme solution onto the biosensor electrodes using a 10 μL pipette connected to the tip (Microloader^TM^, Eppendorf, Germany) that is advantageous for small volume application. In the enzyme solutions for each biosensor, 2,000 U mL^−1^ glucose oxidase, 100 U mL^−1^ lactate oxidase, 100 U mL^−1^ choline oxidase, and 100 U mL^−1^ glutamate oxidase selectively were contained in the BSA solution. BSA solution was composed of 20 mg mL^−1^ BSA, 0.2% GA, and 1% glycerol in 0.1 M PBS, pH 7.4. After adding the enzyme solution, we waited for 1 h at room temperature to sufficiently cross-linking the enzyme layers and removed a PDMS well array. Finally, we completed the enzyme layer–coated biosensors by carefully rinsing the enzyme layer with DI water.

### Characterization of the Integrated Biosensors.

We characterized the biosensors within the fluid flow for in vivo experiments. In any neurochemicals measurement experiments, we applied the working potential of 0.6 V vs. an Ag/AgCl reference electrode, the interval of 0.5 s by chronoamperometry of multiple-channels potentiostats (STAT4000, Metrohm DropSens, Spain). We immersed the shank of the neural probe in a beaker filled with stirred 20 mL of aCSF and applied negative pressure to the extraction tube.

First, to characterize the sensitivity of the biosensor as a function of the extraction flow rate, we set the starting extraction flow rate to 50 nL min^−1^ and then injected 40 μL of 10 mM glutamate into a beaker. Subsequently, the extraction flow rate was increased by increasing the negative pressure step-by-step until it reached a maximum flow rate of 800 nL min^−1^ and then lowered to 50 nL min^−1^ to demonstrate the correlation between flow rate and sensitivity.

Next, an experiment on the crosstalk between biosensors in series-structure microchannels was conducted. The neural probe with a PDMS interface chip carved in a series structure of chambers over biosensors was prepared. From the extracted solution, the first-reacted working electrode was coated with glucose oxidase and the second-reacted working electrode was coated with BSA. After maintaining the extraction flow rate at 100 nL min^−1^, 20 μL of 500 mM glucose was injected into aCSF to measure the crosstalk between the two electrodes in the serial structure of the channel.

Finally, we confirmed the characteristics of biosensors at 100 nL min^−1^ of an extraction flow rate as functions of the concentration of neurochemicals. First, we injected 10 μL of 20 mM DA and 20 μL of 200 mM AA, acting as electroactive interferents, into the aCSF in a beaker. Then, 10 μL of 10 mM glutamate, 20 μL of 500 mM glucose, 10 μL of 20 mM choline, and 20 μL of 500 mM lactate were sequentially injected into the solution. Subsequently, stock solutions of neurochemicals were additionally injected into the solution in a beaker to increase the concentration of neurochemicals. To confirm the reliability of the biosensors, the same packaging method and experimental protocol were performed twice more and the sensitivity of the biosensor was plotted as a graph. Response time of biosensors was defined as 90% of average saturation current compared to before glucose injection, and the detection limit was 3 times average background noise. The plots of all data about biosensor characterization and calculation of the sensitivity and linearity of the biosensor were performed using a statistical analysis program (Prism, GraphPad Software Inc., USA).

## Supplementary Material

Appendix 01 (PDF)

## Data Availability

All study data are included in the article and/or *SI Appendix*.
