# Supplementary material for: A neural probe for concurrent real-time measurement of multiple neurochemicals with electrophysiology in multiple brain regions in vivo
Source: Proc Natl Acad Sci U S A. 2023 Jul 3;120(28):e2219231120. doi: 10.1073/pnas.2219231120 (PMC10334791; doi:10.1073/pnas.2219231120)
Supplement: Supplementary file 1 — Appendix 01 (PDF) [file pnas.2219231120.sapp.pdf]

PNAS

Supporting Information for

A neural probe for concurrent real-time measurement of multiple neurochemicals with electrophysiology in multi brain regions *in vivo*

Uikyu Chae, Jiwan Woo, Yakdol Cho, Jeong-Kyu Han, Soo Hyun Yang, Esther Yang, Hyogeun Shin, Hyun Kim, Hyun-Yong Yu, C. Justin Lee, and Il-Joo Cho*

*Corresponding author: I.-J. C. (ijcho@korea.ac.kr)

This PDF file includes:

Supporting text S1 – S8
Figure S1 to S16
SI References

Supporting text S1 – S8

Methods

S1. Fabrication of the RTBM MEMS neural probe

Our RTBM MEMS neural probe was fabricated with a microfabrication process similar to the previously reported probe(1). We selected an SOI wafer of 40 μm top silicon and formed trenches for integrating microfluidic channels through deep reactive-ion etching (DRIE) processes on the top silicon layer. The trenches consisted of a center cavity (25-μm high and 30-μm wide) for the glass layer anchoring and two sets of five cavities (25-μm high and 6-μm wide) for microfluidic channels located on both sides based on the center cavity. Then, the microchannels were formed through a glass reflow process.

We patterned the gold (Au) signal line (20-nm-thick titanium (Ti) and 300-nm-thick Au) on the silicon substrate with microfluidic channels to electrically connect the electrical and chemical signals. Afterward, 400-nm-thick SiO_2 was deposited on the surface of the wafer using plasma-enhanced chemical vapor deposition (PECVD). The reactive-ion etching (RIE) process was performed to remove the SiO_2 layer at the position where the pads for wire-bonding and the platinum (Pt) electrodes (i.e., recording and biosensor electrodes) would be formed. The recording and biosensor electrodes were deposited with 20-nm-thick Ti and 150-nm-thick Pt using a sputter and defined by a lift-off process. Finally, to define the shape of the probe, the top and bottom of the sample were etched through the DRIE process and a neural probe was released.

To fabricate the PMDS-based interface chip, we first fabricated a master mold via the SU-8 patterning process, the previously reported photolithography method(2). We patterned a 12-μm thick SU-8 layer (SU-8 3010, Kayaku Advanced Materials Inc., USA) as the first layer with multiple drug delivery channels and channel resistance patterns in the extraction channel. Afterward, a 60-μm thick SU-8 layer was secondly patterned in the extraction channel except for the resistance pattern. Finally, the wafer with patterns of microfluidic channels was cut according to the size of the PDMS interface chip with a laser cutter.

S2. Packaging of the RTBM neural probe

We packaged the RTBM neural probe to provide the fluid and electrical interface with external instruments for performing multiple functionalities (i.e., real-time bimodal monitoring and chemical stimulation). First, we attached the fabricated neural probe to a PCB and conducted wire-bonding between the pads of the probe body and of the PCB to provide electrical connections. Then, we applied thermal epoxy to part of the bonded wires and pads and cured the device in an oven at 80 °C for 30 minutes. Afterward, we completed enzyme layer patterning on the working electrode array described above. Additionally, we fabricated the interface PDMS chip that the mixed PDMS with the base to curing agent ratio of 10:1 was poured into a duralumin master mold covered with a piece of silicon with SU-8 microfluidic channels pattern and cured thoroughly at 80 °C for 2 hours. Then, we punched the ports where the tubes were to be connected with a custom punch (inner diameter (ID) of 0.3 mm and an outer diameter (OD) of 0.7 mm). Finally, we applied O2 plasma bonding (100 W, 50 mTorr for 40 seconds) between the fabricated PDMS interface chip and the body of probe with an enzyme-coated biosensor array.

S3. Pt black electroplating

We prepared an electroplating solution by adding 30 mg · ml^{-1} of chloroplatinic acid hydrate, 25 μM HCl, and 0.25 mg · ml^{-1} in DI water according to the previously reported protocol(1). Then, we immersed the recording electrodes (working electrode) of the neural probe and an Ag/AgCl wire (reference electrode) in the electroplating solution. Pt black was electroplated by applying -0.2 V to the recording electrode for 35 seconds to reference the Ag/AgCl wire using the chronoamperometry method of the two-electrode system (PalmSens3, PalmSens, Netherlands).

S4. Characterization of the microfluidic system

We made customized fluidic adapters by plugging pre-cut 23-gauge needles into the Tygon Microbore tubes (ID: 0.05 cm, OD: 0.15 cm). Then, we connected the front end of the customized fluidic adapters to the PDMS interface chip port and the back end of the customized fluidic adapters to the external pressure controllers. We used mass flow controllers (ITV0090-3BL and ITV0051-3BL, SMC Corporation, Japan) to precisely regulate the pressure. Flow controllers require both positive and negative

pressure to operate. The positive pressure was applied to the nitrogen gas tank and the negative pressure was applied to the vacuum pump.

We immersed the shank of the neural probe in 0.1 M PBS and measured the flow rate by precisely controlling the pressure to notice the positive and negative pressure at which the sampling flow rate was 100 nl·min^{-1}. Thereafter, an experiment to confirm the time when the drug and the aCSF solution were switched was performed in the same manner as previously reported(1). Briefly, two delivery ports were filled with red- and blue-dye-infused water, and then the pressure was controlled so that the red dye was injected into PBS for 5 minutes. Afterward, the time at which the blue dye was observed at the probe tip was measured by switching the pressure applied to two ports.

S5. Brain slice preparation and ex vivo experimental procedure

Mouse was anesthetized with isoflurane and decapitated. The brain was then immediately removed and placed in ice-cold slice cutting solution (0–4 °C) containing the following aCSF: 250 mM sucrose, 26 mM $NaHCO_3$, 11 mM KCl, 1.2 mM NaH_2PO_4, 7 mM $MgCl_2$, and 0.5 mM $CaCl_2$. Coronal slices of the hippocampus, 300 μm thick, were obtained using a Vibratome (Leica VT1200S; Leica, Germany). For recording purposes, brain slices were transferred to the following aCSF: 126 mM NaCl, 3.5 mM KCl, 1.2 mM NaH_2PO_4, 1.3 mM $MgCl_2$, 2 mM $CaCl_2$, 25 mM $NaHCO_3$, and 11 mM D-glucose, bubbled with a gas mixture of 5% CO_2 / 95% O_2 to maintain a pH of 7.4. The slices were incubated at 28 °C for 30 minutes to recover. All recordings were performed within 6–8 hours from recovery.

To measure the change in neurochemicals induced by the electrical stimuli, current pulses (6-ms long, 50-Hz two-paired monophasic pulses with amplitudes ranging 300 μA) were applied to 1 to 6 recording electrodes combined to the anode and to 7 to 12 recording electrodes combined to the cathode in combination with a stimulus isolator (model DS3, Digimeter Ltd., UK) and a waveform generator (33500B series, Agilent Technologies, USA).

S6. In vivo experimental procedure

The experimental procedures on animals that were approved by the Korea Institute of Science and Technology (KIST), Seoul, Korea, were performed following the ethical standards outlined in the Animal Care and Use Guidelines of KIST. We used adult male mice (C57BL/6, 10–12 weeks of age) for all in vivo experiments, and the in vivo

experimental procedure was similar to that previously reported(1). Briefly, we mounted a mouse anesthetized with urethane (400 mg · kg^{-1}, intraperitoneal injection) in a stereotaxic frame (David Kopf Instruments, USA), incised the scalp, and made small circular holes in the skull by drilling to reach the target location according to Paxinos and Franklin(3). Then, an RTBM neural probe was fixed to the stereotaxic frame so that the probe shank could vertically lower and reach the brain target regions. A pre-amplifier (Intan Technologies, USA, the RHD2000 system acquired at 20 kS · s^{-1} per channel, band-pass filtered at 0.3 kHz–6 kHz for the detection of action potentials) and customized connectors linked three electrodes were connected to the omnetics and biosensing connectors on an RTBM neural probe, and delivery tubes and extraction tubes were connected to the interface chip. Before implanting the probe shank into the brain tissue, we injected the aCSF into the extraction tubes at 100 ml · min^{-1} and simultaneously applied a working potential to the biosensors for 30 minutes to stabilize the biosensors and remove bubbles in the extraction channels. We calculated the concentration of neurochemicals by comparing the stabilized currents with the current measured on in vivo experiment. Next, the probe slowly lowered at a speed of 1 mm · min^{-1} until the probe tips reached the target regions. Subsequently, the sampling flow rates were matched, and the stabilization of electrical signals and biosensors was performed for an additional 30 minutes. Then the in vivo experiment was performed to investigate the functional connectivity and the correlation of electrical and chemical activities. The high KCl solution (100 nl, 100 mM KCl in aCSF) delivered to modulate neural activity was injected over 1 minute at 30-minute intervals and repeated 3 times in total.

S7. Analysis of data in vivo

The analysis of electrical signals starts with sorting electrical neural spikes using previously reported custom MATLAB(4) (Mathworks, USA). We sorted the neural spikes by setting the spike latency to 1 millisecond and negative and positive threshold voltages. We calculated the firing rates by dividing the number of sorted spikes by time and displayed them as heat maps. In the sorted clusters, autocorrelation was calculated through the signals recorded for 60 seconds (2400-2460 seconds) during the second KCl modulation. The significance of the difference in firing rates was evaluated using the Student's paired t-test (Prism, GraphPad Software Inc., USA). The

firing rate values used for the significance of difference were the average firing rates measured at 5 minutes before and after the KCl injection and during the KCl injection each for 1 minute.

In the chemical signal analysis, the concentration of neurochemicals was expressed by substituting the currents with the concentration as functions of the currents measured in the in vitro characteristics. The quantitative value of concentration was obtained by subtracting the background current obtained from the measured saturated current before implanting the probe shank into the brain tissue. The significance of the difference in neurochemicals was also evaluated using the Student's paired t-test. The significance of the difference in concentrations of glutamate, lactate, and choline was calculated as the average of concentrations before and after KCl injection at 5 minutes and during KCl injection at the maximum neurochemical concentration each for 10 seconds. In glucose, the significance of the difference was calculated by the average of the concentrations at 5 minutes before KCl injection, at the minimum concentration during KCl injection, and at the maximum concentration after injection each for 10 seconds. We explored the correlation between neural signals in the mPFC and MD regions by analyzing their cross-correlation. Initially, we determined the firing rate of all recorded neurons at intervals of 30 seconds and calculated the change in their firing rates. We obtained the change in firing rate by subtracting the firing rate of the current event from that of the previous event and dividing the result by the sum of the two firing rates. Then, we multiplied the change in the firing rate of neurons in the MD by the change in the firing rate of neurons in the mPFC to obtain the cross-correlation value. If the neural activity in the MD region responds to the neural activity in the mPFC region, the cross-correlation value will be close to 1. On the contrary, if the neural activity in the MD region does not respond to the neural activity in the mPFC region, the cross-correlation value will be close to 0.

S8. Viral tracing experimental procedure on the mPFC-MD neural circuit

We performed anterograde tracing to confirm the mPFC-MD neural circuit. We conducted the procedure on adult C57BL/6J male mice (8 weeks old) under isoflurane anesthesia (5% induction, 1% maintenance), which were fixed on a stereotactic apparatus (Ultra Precise Mouse Stereotaxic Instruments; Stoelting Co., USA). We made an incision in the scalp and drilled a craniotomy for injecting the virus into the mPFC using a 30-gauge microinjection cannula (P1 Technologies, USA) and an

UltraMicroPump III (World Precision Instruments, USA) at a rate of 0.06 µl per minute. After injecting approximately 0.3 µl virus into the mPFC (coordinates: AP +1.70 mm, ML - 0.35 mm, DV - 1.85 mm from the bregma), we closed the incision with 9-mm autoclips (205016, MikRon Precision, Inc., USA), and administered antibiotics and analgesics to the mice. We allowed the mice to recover from anesthesia and then kept them in their home cages for 2 weeks to allow the AAV to take effect. To identify GFP neurons or terminals, we anesthetized the mice with a mixture of alfaxalone (40 mg $\cdot$ $per^{-1} \cdot kg^{-1}$) : xylazine (10 mg $\cdot$ $per^{-1} \cdot kg^{-1}$) and perfused them with 0.9% saline and then 4% paraformaldehyde 2 weeks after virus injection. After postfixation, we cut the brains into 40-µm-thick coronal sections with a cryotome (CM300, Leica Camera, Germany); the sectioned brain regions encompassed the mPFC or MD. We washed the sections with PBS three times for 5 minutes at room temperature and then incubated them with Hoechst 33342 (H3570, Thermo Fisher Scientific, USA), a blue fluorescent stain that binds double-stranded DNA, at room temperature for 10 minutes. Finally, we immersed the stained sections in mounting solution for 30 minutes at 37 °C and viewed and photographed them using a TCS SP8 dichroic/CS microscope (Leica).

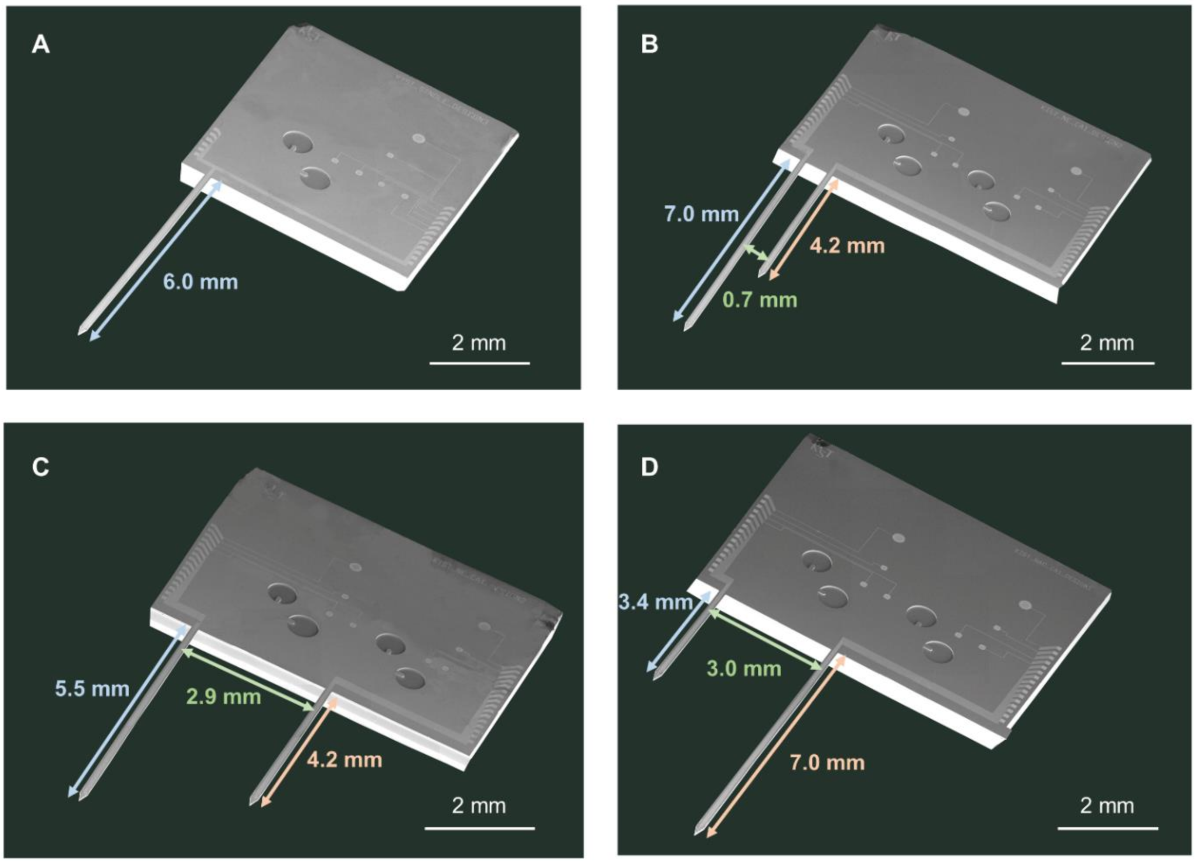

Figure S1. SEM images of various RTBM MEMS neural probes. A, An RTBM MEMS neural probe with a 6 mm shank. **B**, An RTBM MEMS neural probe with two shanks, with a distance of 0.7 mm between the two shanks and shank lengths of 7.0 mm and 4.2 mm. **C**, An RTBM MEMS neural probe with two shanks, with a distance of 2.9 mm between the two shanks and shank lengths of 5.5 mm and 4.2 mm. **D**, An RTBM MEMS neural probe with two shanks, with a distance of 3.0 mm between the two shanks and shank lengths of 3.4 mm and 7.0 mm.

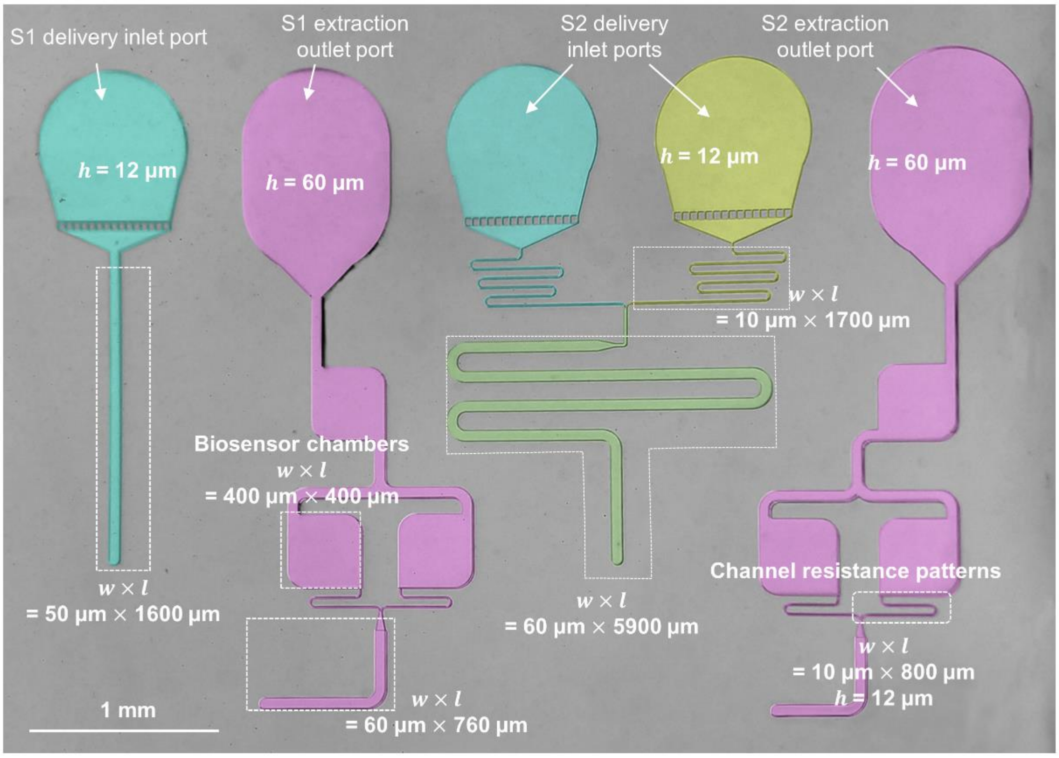

Figure S2. Optical picture of the PDMS-based interface chip for the multi shank RTBM neural probe. The extraction channel (purple) consists of biosensor-compatible chambers, channel resistance patterns, and ports. The delivery channel (green and yellow) consists of two ports for multidrug delivery and a port channel for push-pull perfusion.

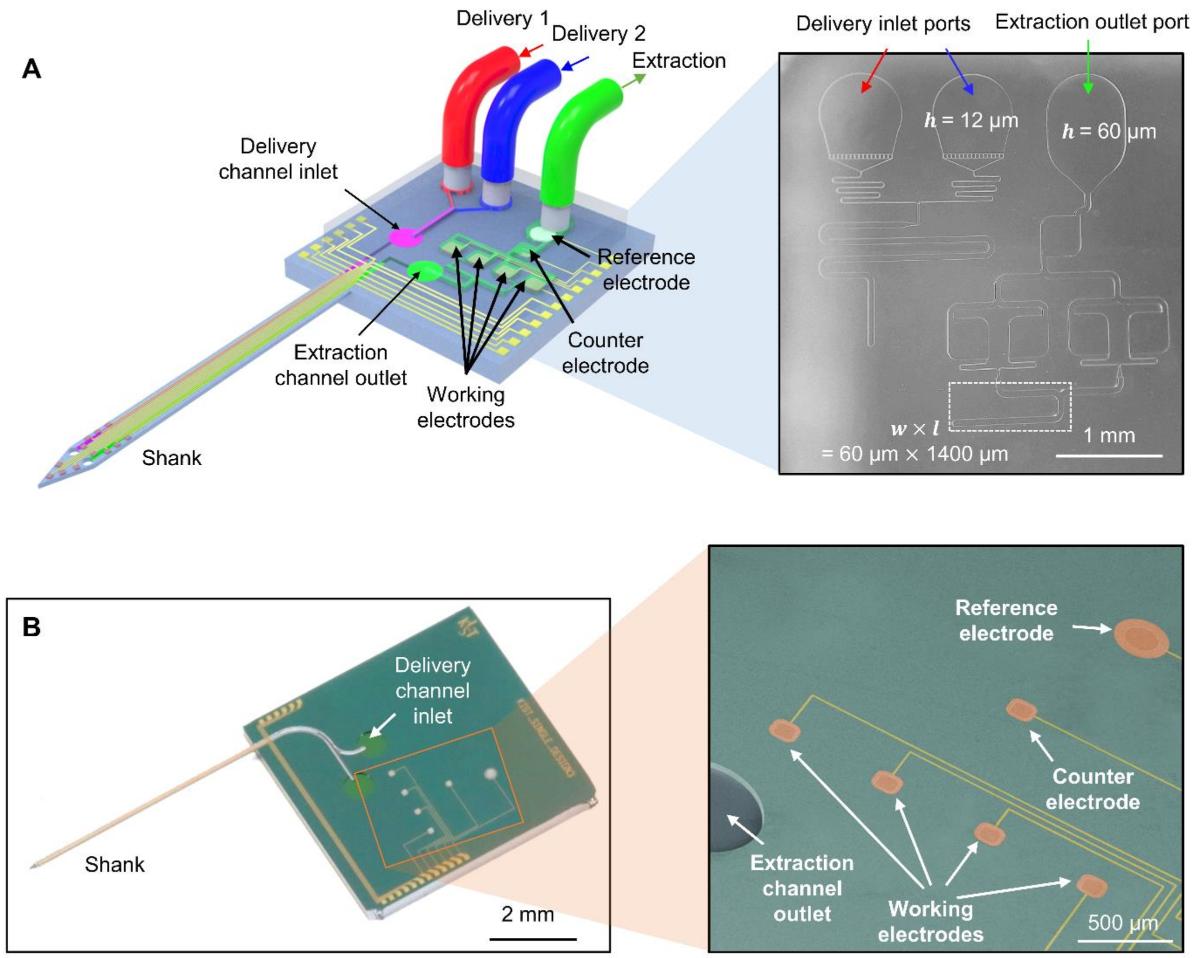

Figure S3. Schematic illustration of the fabrication of the one shank RTBM MEMS neural probe. A, Schematic diagram of the one-shank RTBM MEMS neural probe, which consists of one shank, a probe body with four biosensors and an interface chip with an inset optical image of the fabricated PDMS-based interface chip. **B**, Optical picture of the one-shank RTBM MEMS neural probe and an SEM image of the biosensors on the body.

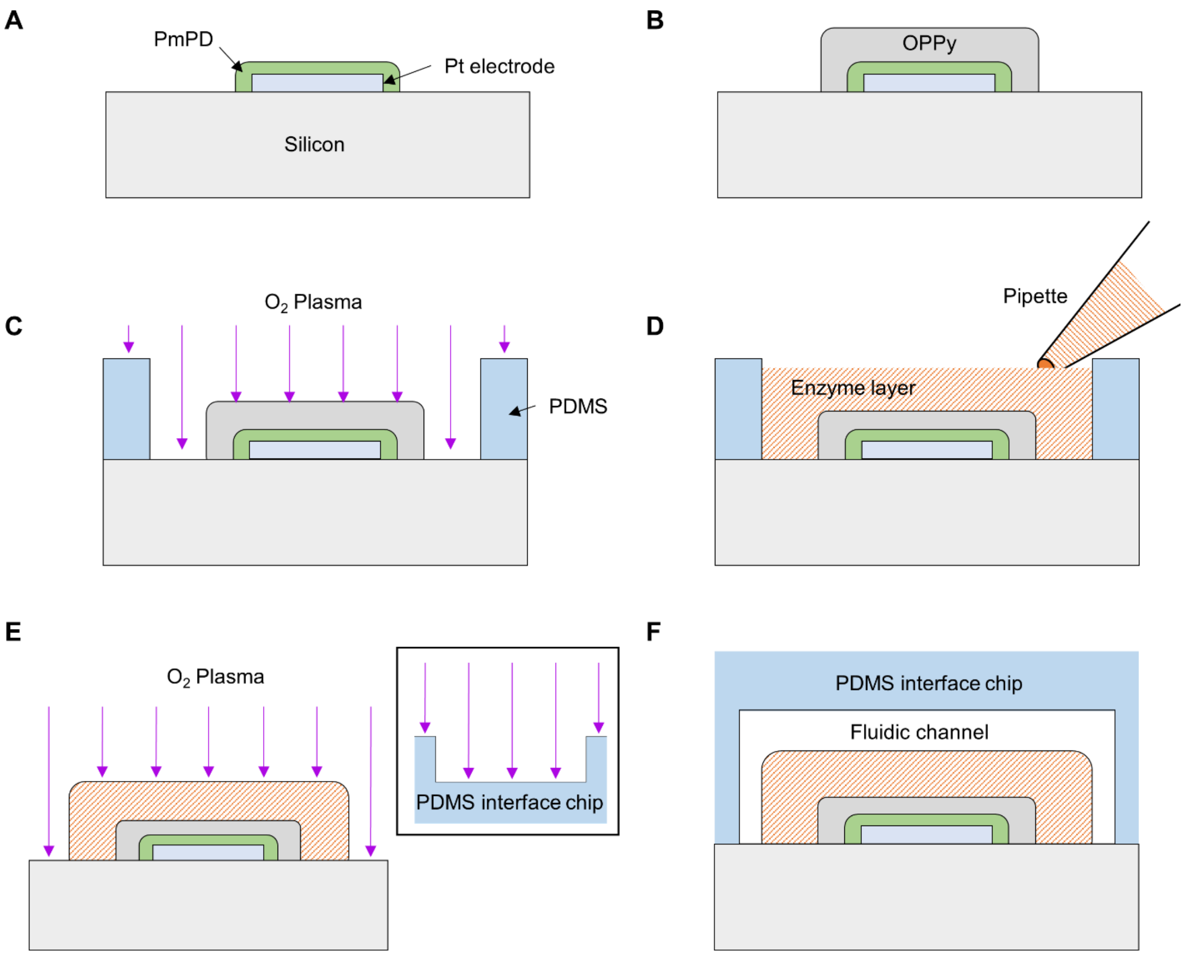

Figure S4. Preparation process of biosensors coated with an enzyme layer. A, Formation of PmPD on a Pt electrode by electropolymerization. **B**, Formation of OPPy on PmPD via electropolymerization and overoxidation. **C**, Alignment of PDMS-based mold produced by laser cutting on biosensors and treatment of the sample with O_2 plasma. **D**, Dropping mixed enzyme solution on the biosensor and cross-linking at room temperature. **E**, Removal of PDMS-based mold and treatment of biosensors with coated enzyme layers and an interface chip with O_2 plasma. **F**, O_2 plasma bonding of an interface chip to the probe body.

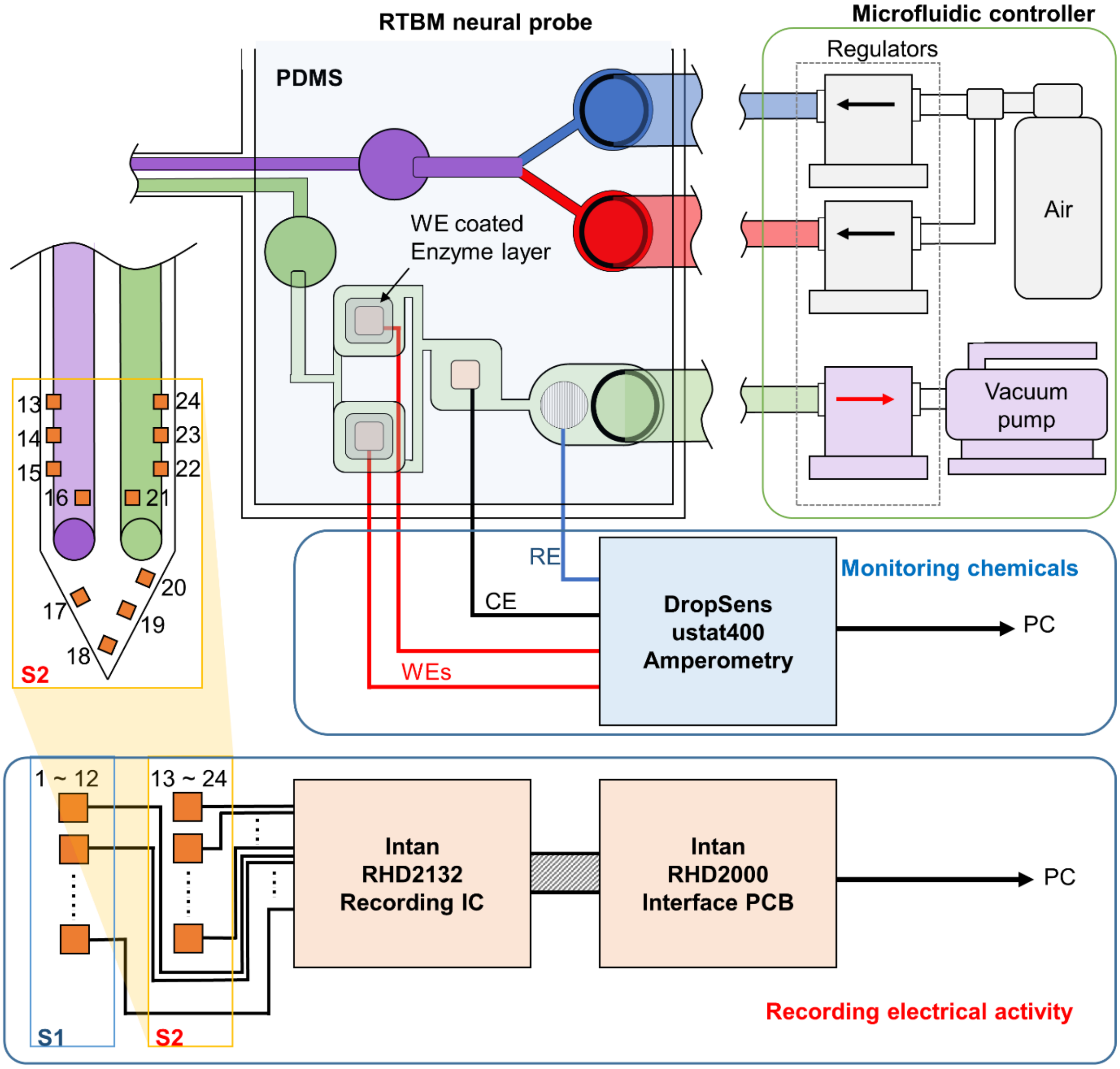

Figure S5. Schematic illustration of the system for the simultaneous observation of multiple neurochemicals and electrical signals. An Air pump and a vacuum pump are provided to regulators as positive and negative pressure sources to deliver drugs and aCSF and to extract ECF at an accurate flow rate. The biosensors measure the neurochemicals contained in the ECF in real time by amperometry of the three-electrode system. At the same time, the electrode placed on the probe tip is electrically connected to the amplifier and interface, and the electrical activity of the neurons is measured and transmitted to the PC.

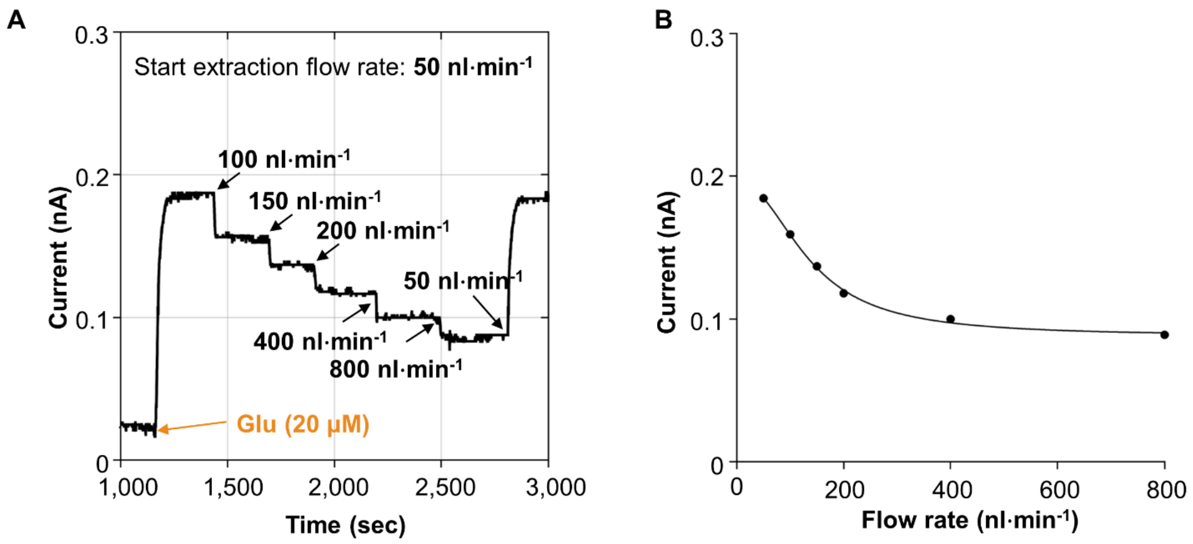

Figure S6. Flow rate dependence on sensitivity. A, Current-time plot as a function of the extraction flow rate. **B**, A calibration curve of the sensitivity as a function of the extraction flow rate.

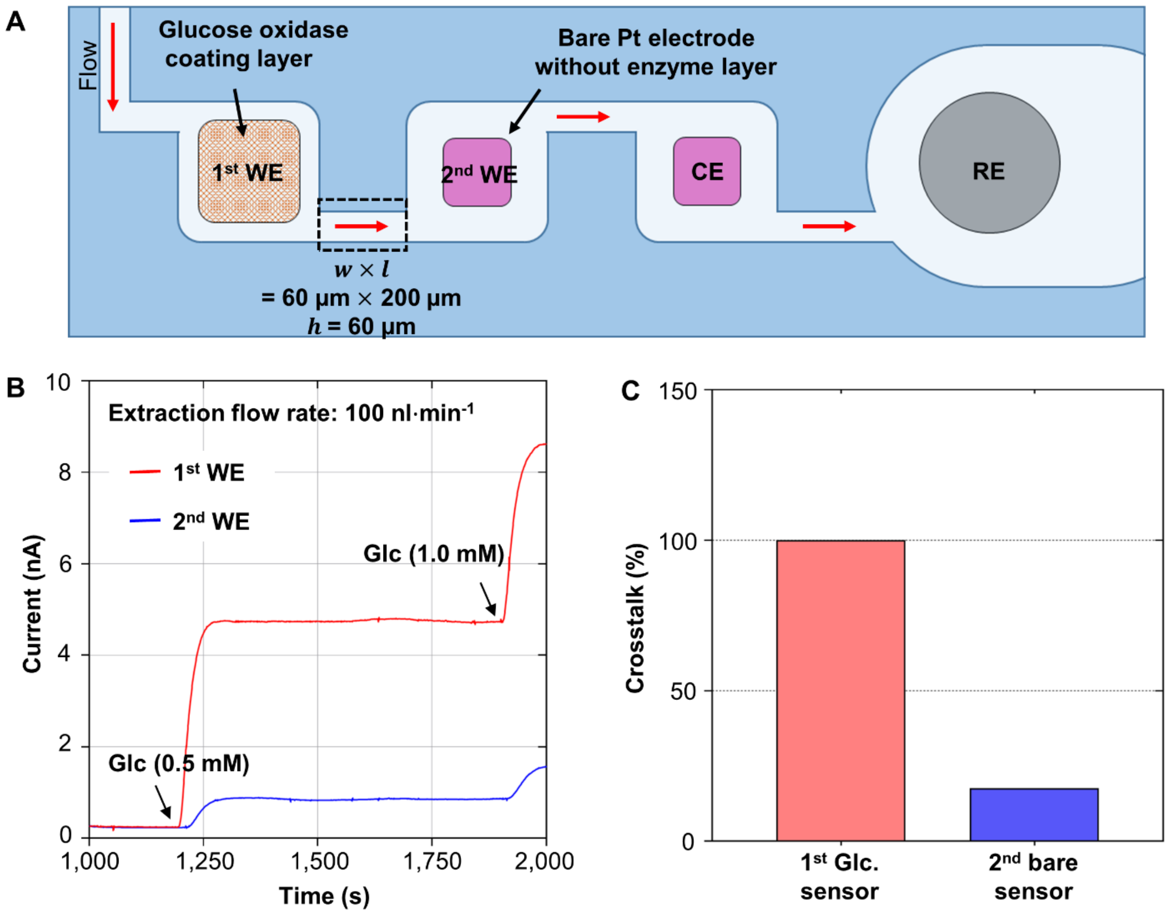

Figure S7. Crosstalk in the serial structure of the channels. A, Schematic illustrating the serial channel structure for crosstalk verification. **B**, Current-time plots as a function of glucose concentration on the 1st WE and 2nd WE. **C**, Comparison of crosstalk of the 1st and 2nd sensors. (The average current measured at the 1st WE with 0.5 mM glucose was normalized to 100% crosstalk.)

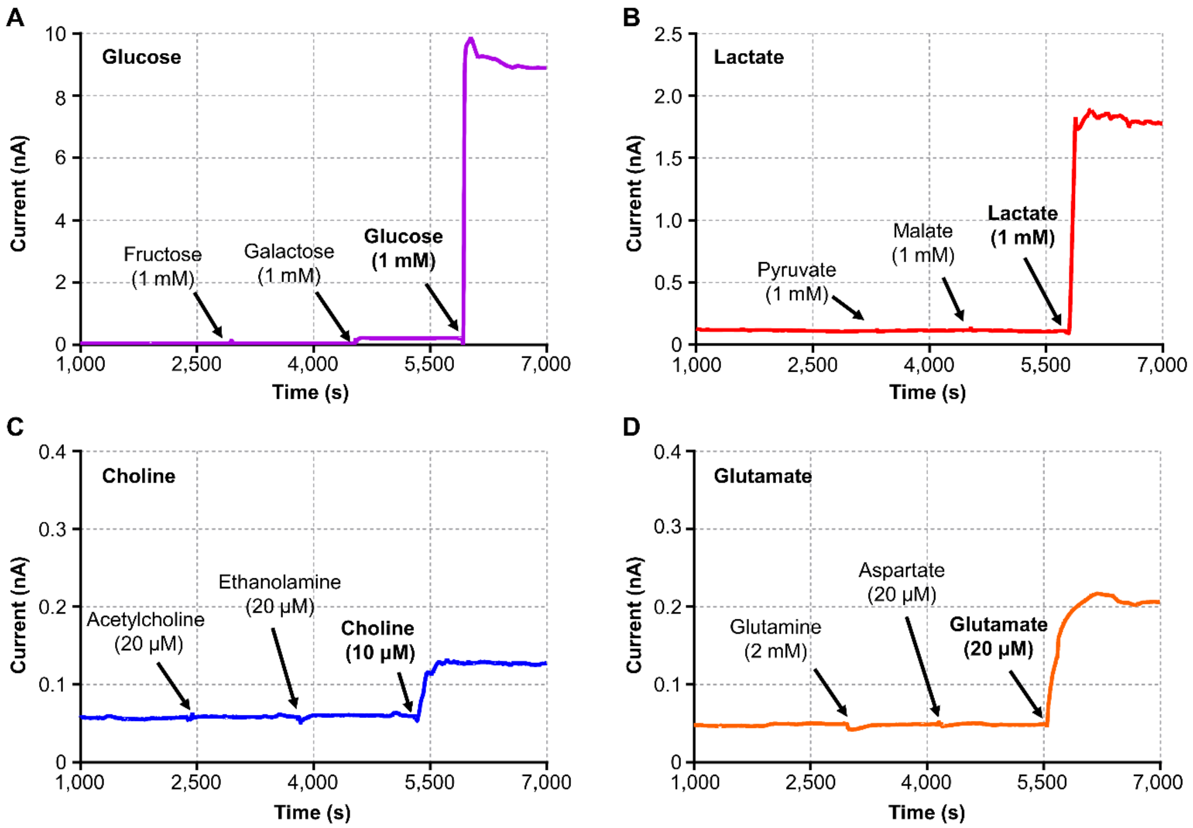

Figure S8. Cross-talk of neurochemicals with similar chemical structures to target neurotransmitters. A, Oxidation current responses of the glucose biosensor to fructose, galactose and glucose. **B**, Oxidation current responses of the lactate biosensor to pyruvate, malate, and lactate. **C**, Oxidation current responses of the choline biosensor to acetylcholine, ethanolamine, and choline. **D**, Oxidation current responses of the glutamate biosensor to glutamine, aspartate, and glutamate.

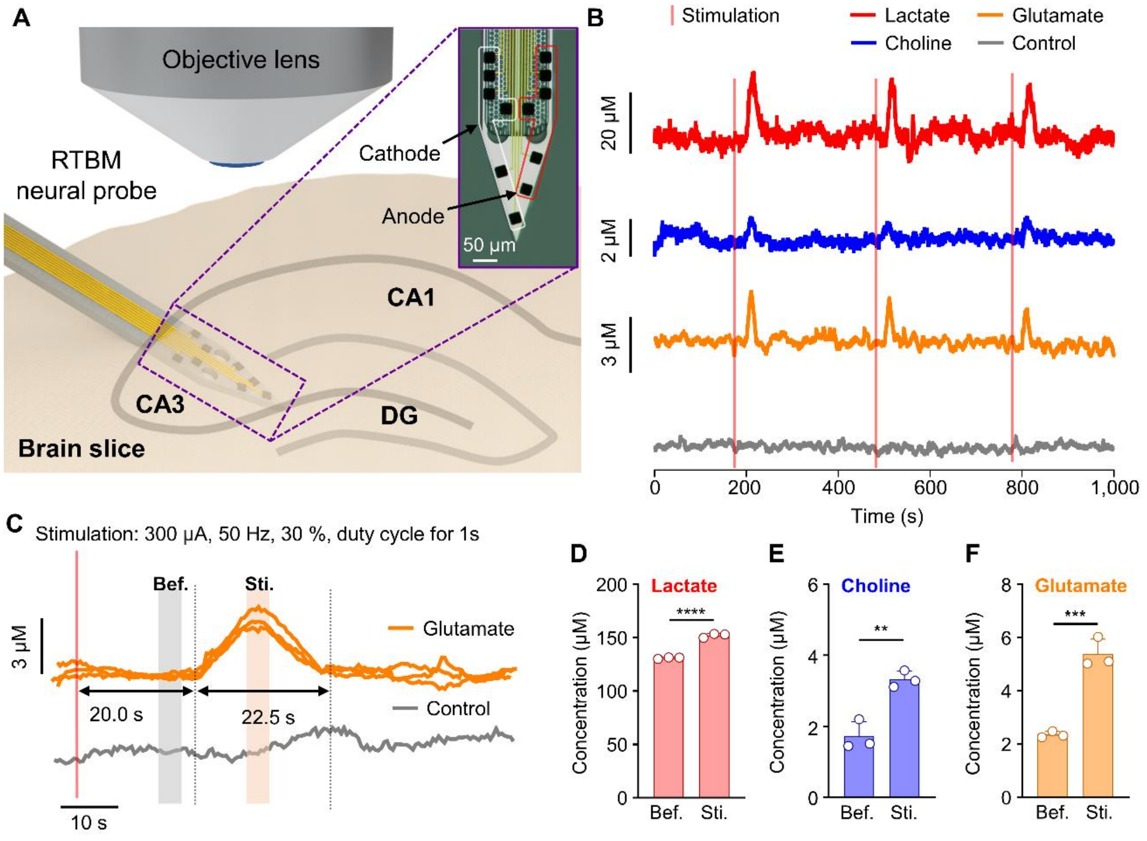

Figure S9. Ex vivo experimental results for real-time monitoring of biosensors integrated with the RTBM MEMS neural probe. **A**, Schematic position of the probe shank and an objective lens in incubation chamber for monitoring neurochemicals from the brain slice with pictures of the recording electrode used as the stimulating electrode anode and cathode. **B**, Response curves showing the concentrations of lactate, choline, and glutamate upon electrical stimulation. **C**, Variation of the concentration of glutamate changed by three times of electrical stimulation. **D-F**, Changes in concentrations of neurochemicals measured before and after electrical stimulation: (**D**) lactate, (**E**) choline, and (**F**) glutamate.

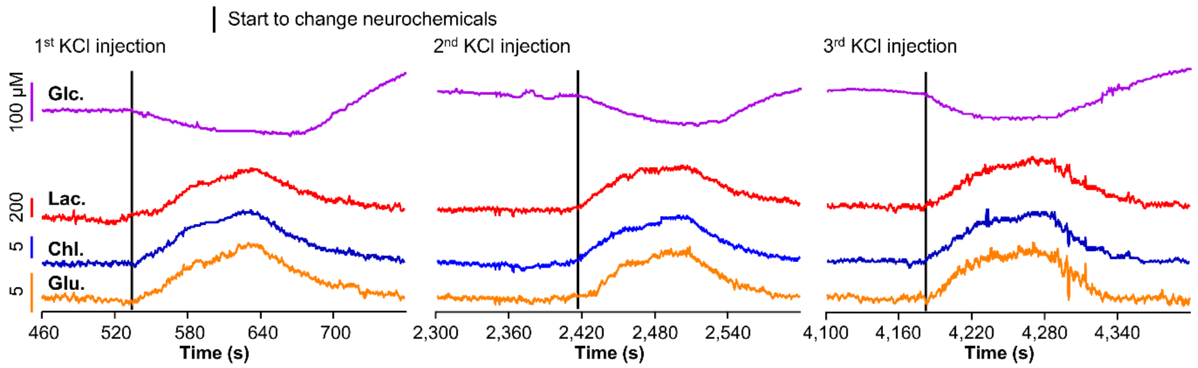

Figure S10. Concentrations of neurochemicals following KCl injection. 1st KCl injection, 2nd KCl injection, and 3rd KCl injection.

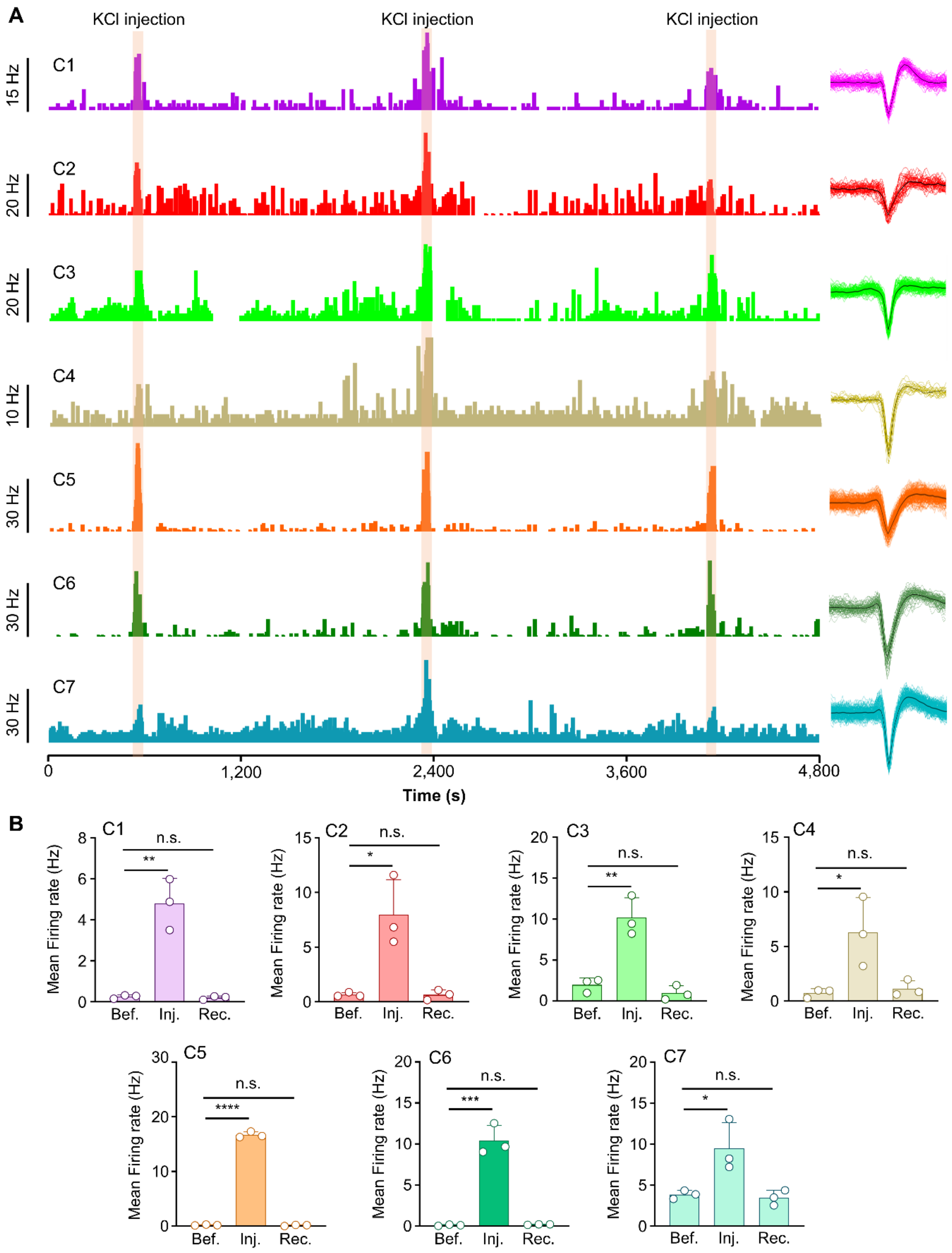

Figure S11. Comparison of the firing rate of electrical activities when high KCl is injected before, during, and after from *in vivo* experiment. A, Firing rates of the sorted neural signals during the total of *in vivo* experiment. **B**, Comparisons of the firing rates of the recorded neural signals from individual neural spikes. The data are

presented as mean ± s.d. (n = 3 for all, Two-tailed t-test). In C1, **P = 0.0032, t = 6.302 (Bef. – Inj.); *n.s.* = 0.5257, t = 0.6944 (Bef. – Aft.). In C2, *P = 0.0170, t = 3.941 (Bef. – Inj.); *n.s.* = 0.9712, t = 0.03848 (Bef. – Aft.). In C3, **P = 0.0051, t = 5.555 (Bef. – Inj.); *n.s.* = 0.2317, t = 1.409 (Bef. – Aft.). In C4, **P = 0.0393, t = 3.015 (Bef. – Inj.); *n.s.* = 0.4398, t = 0.8570 (Bef. – Aft.). In C5, ****P < 0.0001, t = 51.87 (Bef. – Inj.); *n.s.* = 0.4629, t = 0.8109 (Bef. – Aft.). In C6, **P = 0.0007, t = 9.591 (Bef. – Inj.); *n.s.* = 0.1493, t = 1.782 (Bef. – Aft.). In C7, *P = 0.0360, t = 3.105 (Bef. – Inj.); *n.s.* = 0.5665, t = 0.6240 (Bef. – Aft.).

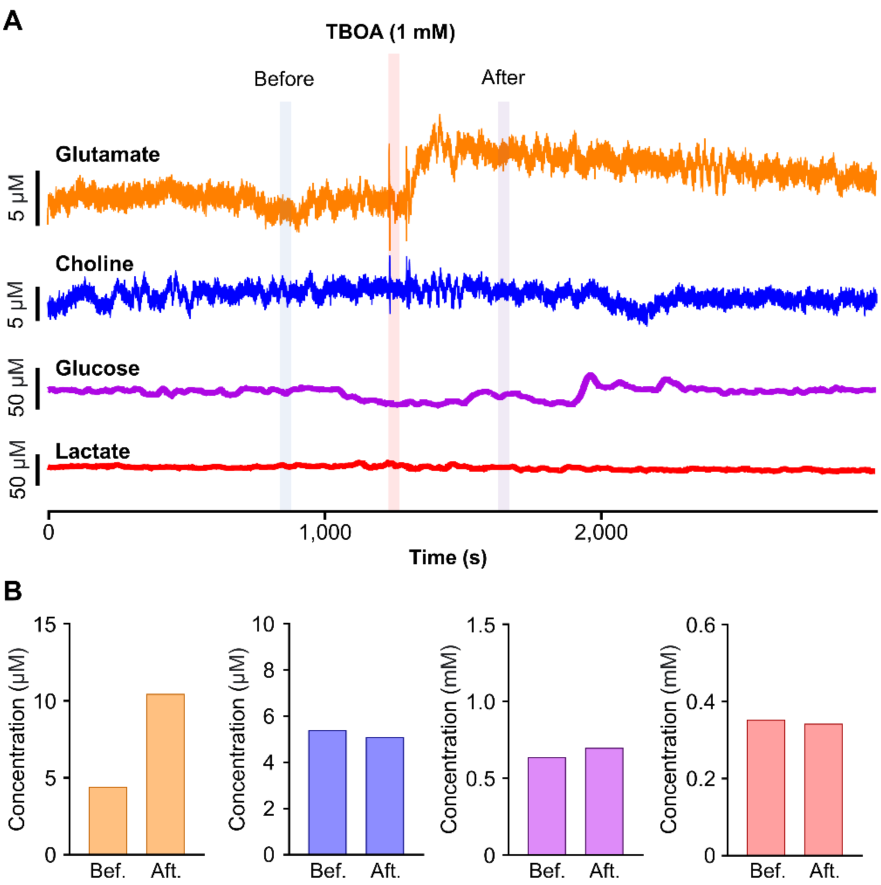

Figure S12. ***In vivo*** **experimental results of selective glutamate modulation with a one-shank RTBM MEMS neural probe for real-time monitoring of four neurochemicals.** **A**, Response curves showing the concentrations of four neurochemicals (glucose, lactate, choline, and glutamate) upon TBOA injection. **B**, Concentrations of each neurochemical at time points 'Before' and 'After' indicated in (**A**).

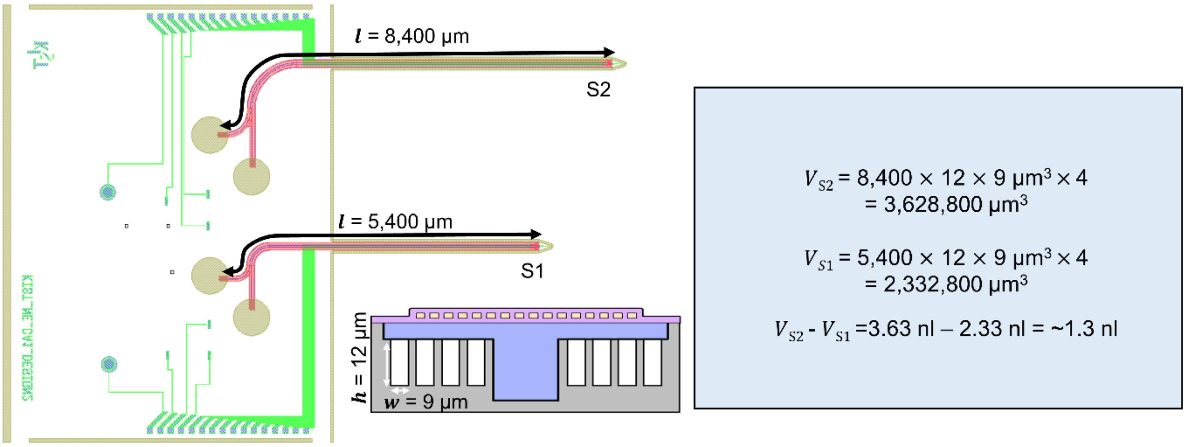

Figure S13. Estimation of microfluidic channel volume of our multi-shank RTBM neural probe. The width (w), height (h), and length (l) of each branch of the fluidic channels in the neural probe are shown. V_{S1} and V_{S2} are fluidic volume of the delivery and collection channels in the shank of the probe implanted into the mPFC and MD, respectively.

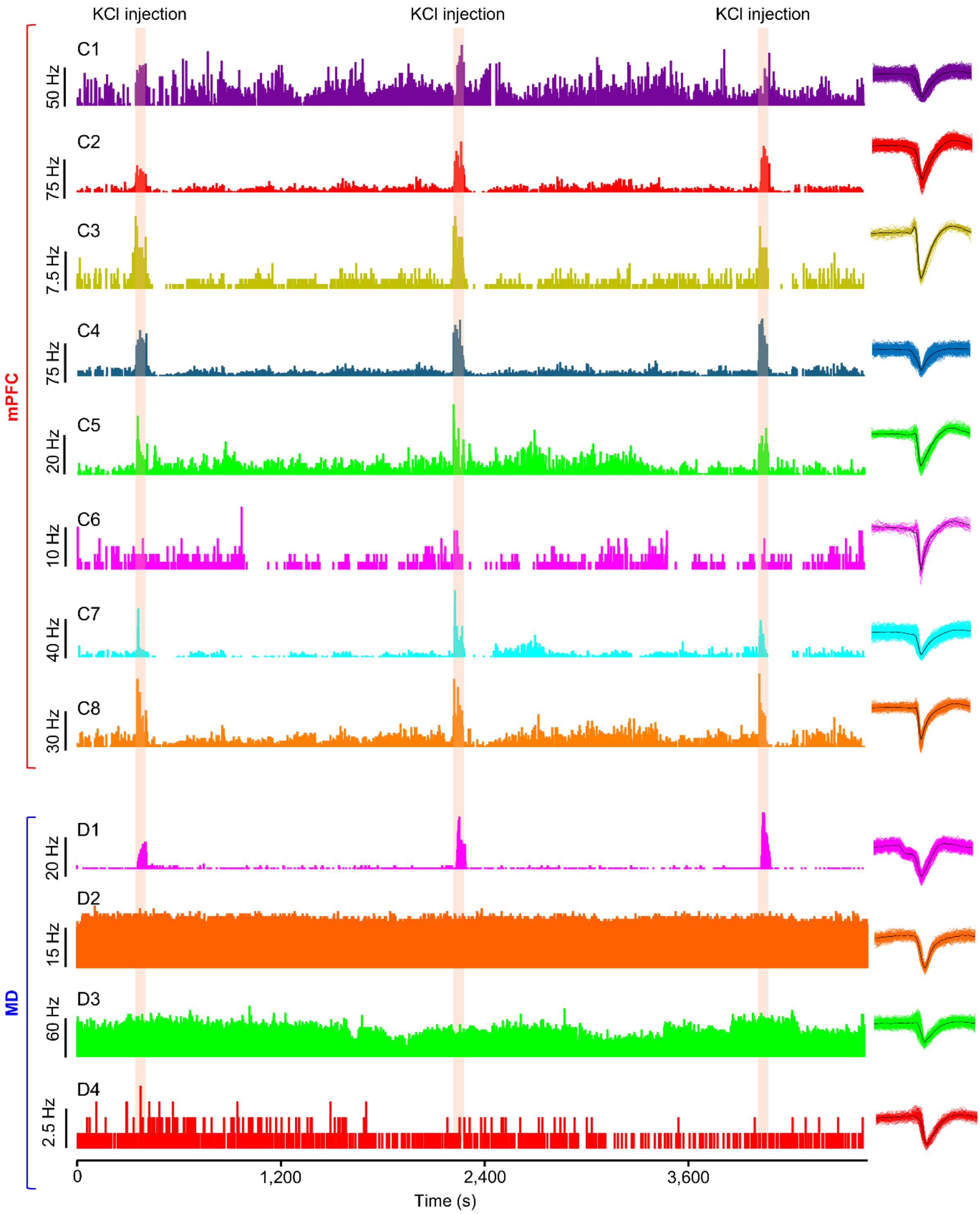

Figure S14. Firing rates of the sorted neural signals in the mPFC and MD during the total of *in vivo* experiment.

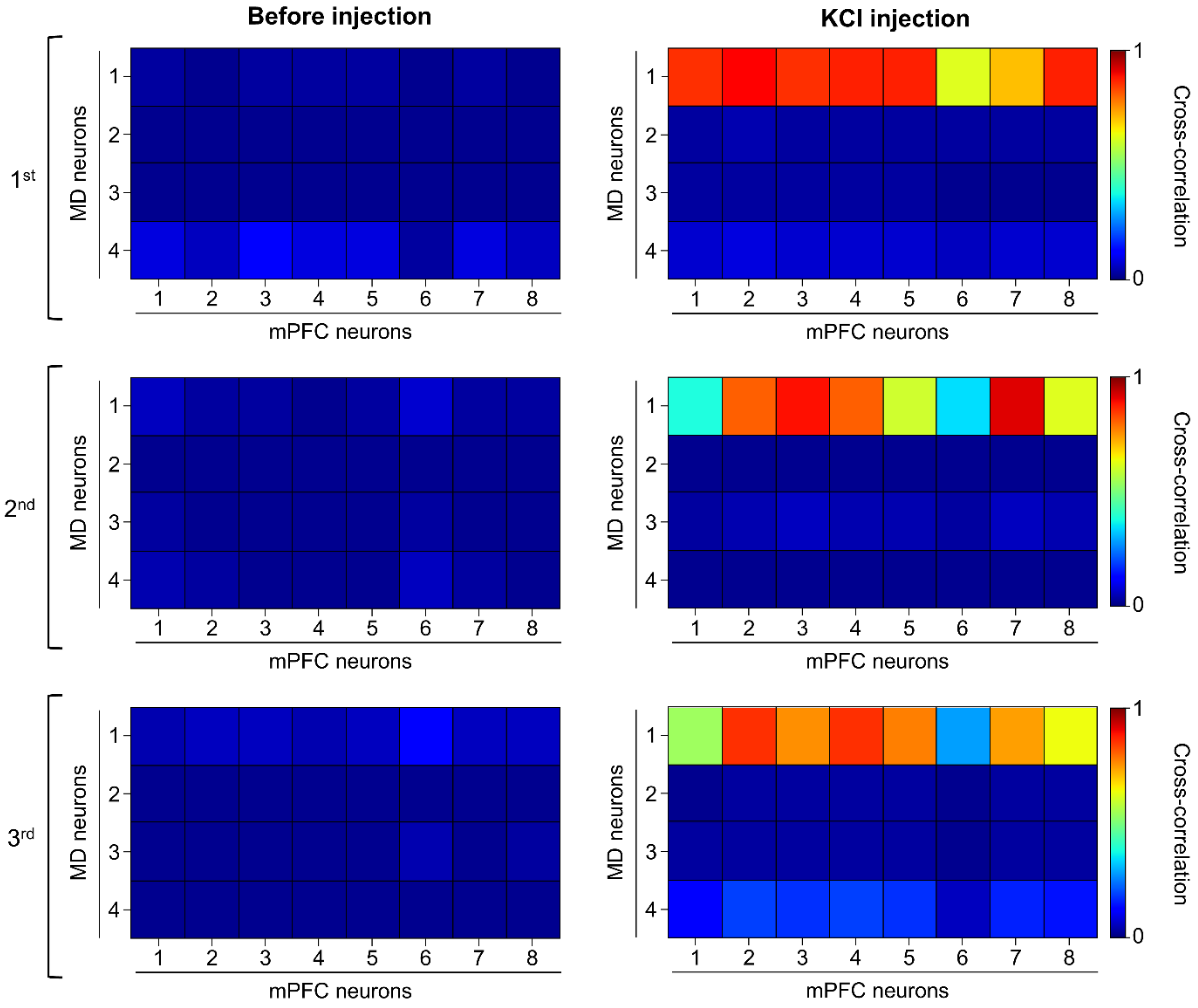

Figure S15. Cross-correlation matrices displaying changes of event detected between neural signals in the mPFC and MD. Color-mapped raster plots showing neural activity before and during three injections of KCl.

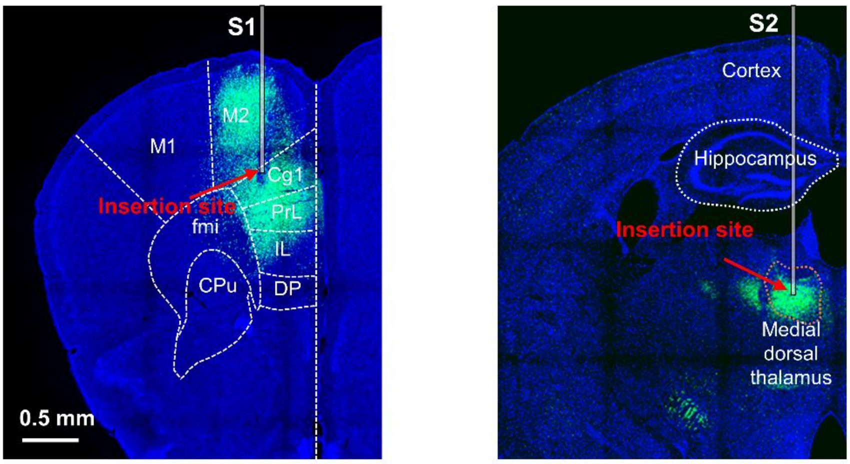

Figure S16. Fluorescence image of brain slices depicting virus tracing from pAAV-U6-GFP injected into the mPFC (Cg1) and projected to the MD region, with markers indicating the sites of probe insertion.

SI References

1. U. Chae *et al.*, Bimodal neural probe for highly co-localized chemical and electrical monitoring of neural activities in vivo. *Biosensors and Bioelectronics* **191**, 113473 (2021).
2. H. Shin *et al.*, Neural probes with multi-drug delivery capability. *Lab on a Chip* **15**, 3730-3737 (2015).
3. G. Paxinos, K. B. Franklin, *Paxinos and Franklin's the mouse brain in stereotaxic coordinates* (Academic press, 2019).
4. H. Shin *et al.*, Multifunctional multi-shank neural probe for investigating and modulating long-range neural circuits in vivo. *nature communications* **10**, 1-11 (2019).
